# Practices for Research Integrity Promotion in Research Performing Organisations and Research Funding Organisations: A Scoping Review

**DOI:** 10.1007/s11948-021-00281-1

**Published:** 2021-01-27

**Authors:** Rea Ščepanović, Krishma Labib, Ivan Buljan, Joeri Tijdink, Ana Marušić

**Affiliations:** 1 School of Medicine, Department of Research in Biomedicine and Health, University of Split, Split, Croatia; 2 Department of Ethics, Law and Humanities, Amsterdam University Medical Centers, Amsterdam Public Health Institute, Vrije Universiteit Amsterdam, Amsterdam, The Netherlands; 3 Department of Philosophy, Vrije Universiteit Amsterdam, Amsterdam, The Netherlands

**Keywords:** Research integrity, Research ethics, Research integrity practices, Research integrity promotion, Research performing organisations, Research funding organisations

## Abstract

Research integrity (RI) is a continuously developing concept, and increasing emphasis is put on creating RI promotion practices. This study aimed to map the existing RI guidance documents at research performing organisations (RPOs) and research funding organisations (RFOs). A search of bibliographic databases and grey literature sources was performed, and retrieved documents were screened for eligibility. The search of bibliographical databases and reference lists of selected articles identified a total of 92 documents while the search of grey literature sources identified 118 documents for analysis. The retrieved documents were analysed based on their geographical origin, research field and organisational origin (RPO or RFO) of RI practices, types of guidance presented in them, and target groups to which RI practices are directed. Most of the identified practices were developed for research in general, and are applicable to all research fields (n = 117) and medical sciences (n = 78). They were mostly written in the form of guidelines (n = 136) and targeted researchers (n = 167). A comprehensive search of the existing RI promotion practices showed that initiatives mostly come from RPOs while only a few RI practices originate from RFOs. This study showed that more RI guidance documents are needed for natural sciences, social sciences, and humanities since only a small number of documents was developed specifically for these research fields. The explored documents and the gaps in knowledge identified in this study can be used for further development of RI promotion practices in RPOs and RFOs.

**Supplementary Information:**

The online version contains supplementary material available at 10.1007/s11948-021-00281-1.

## Introduction

The scientific community emphasises the importance of research integrity (RI) because it represents the basis for the advancement of reliable and trustworthy knowledge and scientific endeavours (Aubert Bonn et al. 2017). In some countries, RI is also referred to as responsible conduct of research (RCR) (DuBois 2004; Steneck 2006; Kalichman 2013; Shamoo and Resnik 2015). In addition to providing written guidance for good research and mechanisms to encourage compliance with responsible practices, RI is also an integral part of researchers’ moral obligation to be honest and responsible toward the system of science (Institute of Medicine and National Research Council 2002; Kalichman 2013).

Issues related to RI, RCR, research misconduct, and detrimental (questionable) research practices started to get more attention from the scientific community around the 1990s (Resnik and Shamoo 2017). Initiatives to prevent RI breaches started to develop at the same time, including the development of guidance documents, the examination of their quality in helping researchers to tackle these issues (Nobel, 1990), and the establishment of RI bodies, like the Office of Research Integrity in the USA (Steneck 2006). Whereas at the beginning of the development of RI as a field, the focus was on the individual researchers and prevention of misconduct, the promotion of RI and prevention of misconduct are today seen as a mutual responsibility of different organisations and individuals included in research (NASEM 2017, Bouter 2018; Hermerén 2019). Better understanding of RI and its implementation in practice is seen possible only if everyone acts responsibly and accomplishes their tasks related to RI promotion. This includes the responsibility of researchers to conduct research following the good practices and policies provided by research performing organisations (RPOs) and research funding organisations (RFOs). It includes the responsibility of both RPOs and RFOs to implement policies on good research practices, provide education to researchers, and have mechanisms in place that will deal with breaches of RI (Boeheme et al. 2016). Also, the responsibility of journals to prevent poor publication practices that may have detrimental consequences for the scientific community and society in general is important (Marušić et al. 2007; Bouter 2018).

A number of studies have addressed RI issues related to different stakeholders and disciplinary fields. Olesen et al. and Haven et al. explored the research misconduct perceptions of researchers from different disciplinary fields (Olesen et al. 2018; Haven et al. 2019). The effectiveness of existing interventions for RI improvement in different disciplinary fields, such as training and education or implementation of procedures for handling cases of misconduct have also been explored (Marušić et al. 2016). Recently, emphasis has been put on RPOs’ and RFOs’ role in RI promotion. RPOs have an important role in the development and implementation of RI policies and compliance mechanisms (Forsberg et al. 2018). Through its organisational directors and boards, they have a role in raising awareness on RI issues, creating the environment of integrity, and changing the research evaluation practices and incentive structures (Hicks et al. 2015, Boeheme et al. 2016; Moher et al. 2019, Zwart and ter Meulen 2019; Bouter 2020). However, RPOs are not the sole actors in this vital mission, since their efforts in RI promotion can be augmented even more with the endeavours of RFOs (Bouter 2018, 2020). By implementing policies for good research practices and emphasising the importance of RI in funded research, such as the Wellcome Trust in their Guidelines for Good Research Practice (Wellcome Trust 2018), funders can impose high RI standards that need to be respected by those who apply for funds (both individual researchers and research organisations). These may include requests for RPOs to have fair procedures for dealing with RI, requests for researchers to provide a clear explanation of the relevancy of their study, and requests for adequate reporting and open access publishing of the study results to achieve reproducibility of its findings (Begley and Ioannidis 2015; Bouter 2016, NASEM 2018).

As the new knowledge on RI responsibilities of researchers and organisations is emerging, new documents are being developed. However, these documents are scattered through the academic literature, official sites of different RPOs and RFOs, and other professional organisations and networks. Also, RI guidance is presented in various types of documents—codes, guidelines, checklists, standard operating procedures, and others. Although there are studies on the existing RI policies in specific disciplinary fields, as well as research on the diversity of existing policies and terminology used across these documents (Godecharle et al. 2014; Komić et al. 2015; Aubert Bonn et al. 2017), there is no systematic effort to synthesise the knowledge of RI promotion practices in RPOs and RFOs. In this scoping review, we provide a broad overview of the RI guidance documents originating from the scientific literature and grey literature sources. In our analysis, we mapped the documents based on their geographical, disciplinary field and organisational origin, as well as based their relevance for different individuals and organisations in the research process. Our analysis also included identification of different RI topics related to different phases of the research process and the analysis of principles of good research declared in the documents. By exploring these guidance documents and the prescriptive and aspirational norms provided in them, we identified gaps in how RPOs and RFOs address RI and issues in this field that require additional attention.

## Methods

We used a scoping review methodology (Tricco et al. 2016) following the guidance published in the Joanna Briggs Institute (JBI) Review's Manual (Peters et al. 2015).

### Concept and Context

The concept of this review was that there is a wide range of existing practices/guidance documents in RPOs and RFOs with implications on RI promotion and avoiding research misconduct, as well as that these guidance documents may vary in their scope, means of addressing RI issues and stakeholders (e.g. policymakers, researchers, reviewers, students, committees and boards) to which they are directed.

This review examined the practices/guidance documents for RI promotion and avoiding research misconduct related to RPOs, RFOs, and other various stakeholders involved in research (policymakers, researchers, reviewers, students, committees and boards) with the aim of building an overarching view of the current situation regarding RI guidance. Moreover, the review examined RI guidance documents that exist in different research fields and are related to different research phases (research planning, conducting, dissemination and evaluation). It also explored the guiding principles presented across documents, as these principles could serve RPOs and RFOs in creating and preserving the RI environment (NASEM 2017).

### Selection Criteria

The main eligibility criterion for the documents from peer-reviewed journals and grey literature was that these documents addressed any aspect of RI in RPOs and/or RFOs. By any aspect we meant RI issues related to different phases of the research process and with the different RI focus. For example, authorship issues, data management issues, investigations of research misconduct, RI education and other.

A description or summary of RI practices had to be provided in these documents in order for them to be included in the analysis. Editorials and commentaries were included as well when they met the above mentioned criterion.

We included all types of guidance documents on RI issues as ‘practices’. This included guidance in the form of codes, guidelines, checklists, and standard operating procedures but did not exclude other types of guidance documents. Hence, the list of the different forms in which guidance for RI was presented was updated during the process of document screening and analysis.

Although the majority of documents contained the type of guidance on RI issues in their title or description, for documents that were not defined regarding the type of guidance we used the following criteria:

- Code—a document providing general, rather than detailed guidance on ethical standards, principles, values, and rules of behaviour;
- Guideline—a document more specific than code in providing guidance; a document providing specific instructions for performing a certain task or achieving a certain goal;
- Checklist—a document presented as a clear list of items to be done, checked, or considered in performing a specific task;
- Standard operating procedure (SOP) —a document providing detailed, step-by-step instructions for carrying out routine tasks and aimed at achieving uniformity and efficiency;
- Flowchart—a document presenting guidance in the form of a diagram representing a workflow or process;
- Legal document—a document established by a government or other authority, empowered by law, and outlining legal consequences; and
- Policy—a document established and implemented by an organisation, containing adopted principles, rules, and procedures for conducting certain actions.

Other types of guidance used as a category in this review included reports, statements, declarations, white papers, as they had such a term set out in the title or description of the document.

Since academic integrity comprises fundamental values relevant for researchers and their work (Fishman 2014), documents related to academic integrity were included into our analysis whenever they reflected on research performance or researchers’ behaviour, be it professional or unprofessional. Further, documents related to research ethics (RE) were also included if they addressed issues similar to RI, since RE and RI are not always clearly distinguished (Komić et al. 2015).

The search addressed practices relating to different scientific disciplines, categorised in advance as—medical sciences (including biomedicine), natural sciences (including engineering), social sciences, humanities, and ‘research in general’. The latter term was used to map the practices that were not developed for RI in a specific field, but rather to be applicable across different scientific fields.

The search of bibliographic databases did not have geographical or language restrictions, while the grey literature search was limited to documents in English because of the possibility of retrieving a large number of documents that would need to be translated in order to be analysed. Since research misconduct emerged as an important problem in the late 1980s and 1990s (Resnik and Shamoo 2017), only the materials dating from 1990 onward were included in the screening process. The reason for this was based on the need for ensuring applicability and contemporaneity of identified practices and exploring currently existing gaps in knowledge.

### Search of Bibliographical Databases

The search strategy was developed by three researchers who were assisted by a librarian specialised in systematic review search methodology. The development of the search strategy aimed at high sensitivity and included a broad approach to the field, based on the need for the identification of as many relevant documents as possible. As a starting point in the development of the search strategy, we used terms from the European Code of Conduct for Research Integrity (ALLEA 2017). The search strategy is available in Appendix 1 (Electronic Supplementary Material). We searched Scopus, Web of Science (WOS), Medline and PsycINFO bibliographical database. The search of Medline, WOS, and Scopus was performed on 18 February 2019, while the search of PsycINFO was performed on 12 February 2019. The obtained data were exported to the EndNoteTM tool (Clarivate Analytics, Philadelphia, PA, USA).

### Search of Grey Literature Sources

The search of grey literature encompassed several different sources: Open Grey database (Open Grey, INIST-CNRS), World Conferences on Research Integrity (WCRI) (The World Conferences on Research Integrity) website, the Community Research and Development Information Service (CORDIS) database (European Commission), Office of Research Integrity (ORI) (The Office of Research Integrity) website, European Network of Research Integrity Offices (ENRIO) (The European Network of Research Integrity Offices) website, the National Academies of Sciences, Engineering, and Medicine (NASEM) publications (The National Academies of Sciences, Engineering and Medicine), Science Europe publications (Science Europe), Mutual Learning Exercises (MLE) on Research Integrity reports (European Commission), and the League of European Research Universities (LERU) publication (The League of European Research Universities). Details of the search of grey literature sources are presented in Appendix 2 (Electronic Supplementary Material).

### Selection of Documents

For documents that were retrieved by the search of bibliographic databases, duplicates and articles dating before 1990 were first removed and then the screening of the titles and abstracts was performed. The screening was conducted independently by two reviewers. In order to precisely define the criteria and the screening process, as well as to ensure that both reviewers would perform the task in the same manner, the reviewers first performed a pilot screening of the titles and abstracts of 100 records. After the pilot screening, they proceeded with the screening of the titles and abstracts of all the documents, after which they compared and discussed the obtained results in order to decide which documents would be included in the full-text analysis. In cases of disagreement, the final consensus decision was reached after a discussion with the third reviewer. In the following step, the three reviewers performed a full-text assessment of the documents in order to decide whether they were eligible for inclusion into the final analysis. To be included in the final analysis, a consensus had to be reached by at least two reviewers. In cases of major disagreements, the material was discussed with an additional reviewer. Documents that were not written in English were translated using tools such as Google Translate to explore whether they fulfilled the eligibility criteria. Reference lists of the documents included in the final analysis were screened by one reviewer to identify additional documents (sources of practices).

For grey literature sources, one researcher performed the search to identify documents that specifically met the set eligibility criteria. This means that all available documents were not extracted and screened, but rather the full-text screening was performed simultaneously with the search.

### Data Extraction Process

**Fig. 1:**
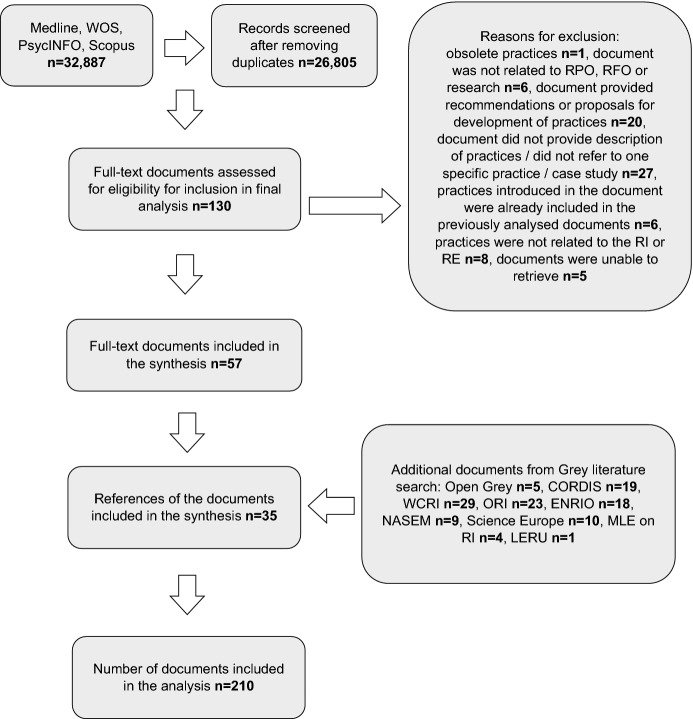
PRISMA-ScR flow diagram for the scoping review process. *CORDIS* Community Research and Development Information Service; *ENRIO* European Network of Research Integrity Offices; *LERU* League of European Research Universities; *MLE on RI* Mutual Learning Exercises on Research Integrity; *NASEM* National Academies of Sciences, Engineering and Medicine; *ORI* Office of Research Integrity; *RE* research ethics; *RI* research integrity; *RFO* research funding organisation; *RPO* research performing organisation; *WCRI* World Conferences on Research Integrity; *WOS* Web of Science

For the documents from the bibliographic databases included in the final analysis (Fig. 1), two researchers performed the data extraction. The list of categories to be extracted was defined in advance and was continually updated by each researcher during the charting process. The list is available in Appendix 3 (Electronic Supplementary Material). The categories were discussed by authors to reach the consensus on the final list. The data extraction of the material obtained from the grey literature search was performed by one researcher.

The following data were extracted: author(s) (for documents from bibliographic databases); title (for documents from bibliographic databases); year of publication; reference type, i.e. journal article, book, book section (for documents from bibliographic databases); journal (for documents from bibliographic databases); country of origin; research fields, i.e. humanities, social sciences, natural sciences (including engineering), medical sciences (including biomedicine), research in general; name of the practice; type of practice (type of guidance on RI issues), i.e. code, guideline, checklist, SOP, legal document, report, declaration, statement, flowchart, white paper, policy; whether the practice was more related to RPOs or RFOs or both; whether the practice was more related to institutions (organisations) or individuals or equally to both; target audience in practice, i.e. researchers, research groups, policymakers, funders, students, mentors and supervisors, committees and members of committees, RI offices and officers, RI advisors, ombudsman, reviewers, administrators, whistle-blowers; description of the source of practice (for grey literature); principles addressed in practices. Documents were also categorised according to the phase of the research process—planning, conducting, dissemination, evaluation—as well as RI violations and resolutions and RI promotion. Within each research process, several RI topics were identified based on their relatedness to the process. Since the main research processes were defined broadly, the grouping of RI topics which were more related to the specific issue enabled us to capture the most prevalent RI issues addressed across practices. Two researchers independently developed the lists of RI topics during the extraction process. After finalising the analysis, the lists of RI topics were compared to detect any overlaps. The list of topics was finalised through a discussion and consensus between two researchers and in consultation with a third researcher.

### Data Synthesis

After the data extraction, all the documents were summarised and analysed based on their geographical origin, the scientific field and organisational (RPO or RFO) origin of the identified practices, the types of practice (the type of guidance), and the target group to which the practices were directed. We also categorised the documents based on the research processes and RI topics addressed in them.

Furthermore, we extracted the guiding RI principles that were explicitly addressed in the documents. This means that RI principles had to be explicitly mentioned and explained in the chapters or parts of the text. The documents just stating RI principles without further elaboration were not included in the analysis. We mapped the extracted principles to the principles presented by the All European Academies in the European Code of Conduct for Research Integrity (ALLEA 2017) and those presented by the US National Academies of Sciences, Engineering, and Medicine in the book Fostering Integrity in Research (NASEM 2017). The aim was to observe the similarity in principles, and terms used to address the guiding principles. We used these two documents because of their wide recognition and acceptance, as well as their up-to-dateness (both were updated in 2017). The extracted principles were mapped by one researcher and checked with the second researcher, upon which the agreement was reached for the final mapping.

## Results

The search of Scopus, WOS, Medline, and PsycINFO retrieved 32,887 documents, 26,805 of which remained after removing the duplicates. The screening of the titles and abstracts left 130 documents for the full-text assessment of eligibility for the final analysis. In the following step, 73 documents were excluded, leaving 57 for the final analysis. The most prevalent reason for exclusion of 73 documents was that the documents did not present actual practices related to research or to RI or RE. Full details on the excluded documents are presented in Fig. 1. Five documents were excluded because we were unable to retrieve them in full text for analysis. The screening of the references from 57 documents included in the final analysis identified additional 35 documents (sources of practices) that were subsequently included in the final analysis and data charting. These additional documents (n = 35) were documents (codes, guidelines, books) provided on the websites of RPOs, RFOs, or other professional organisations. Reference search identified a single additional journal article (a commentary).

The search performed in the Open Grey database, the websites of the World Conferences on Research Integrity, CORDIS, ORI, ENRIO, NASEM, and MLE identified 118 documents that described the practices for the analysis. The total number of all documents included in the final analysis was 210 (Fig. 1).

### Origin of RI Practices

**Fig. 2:**
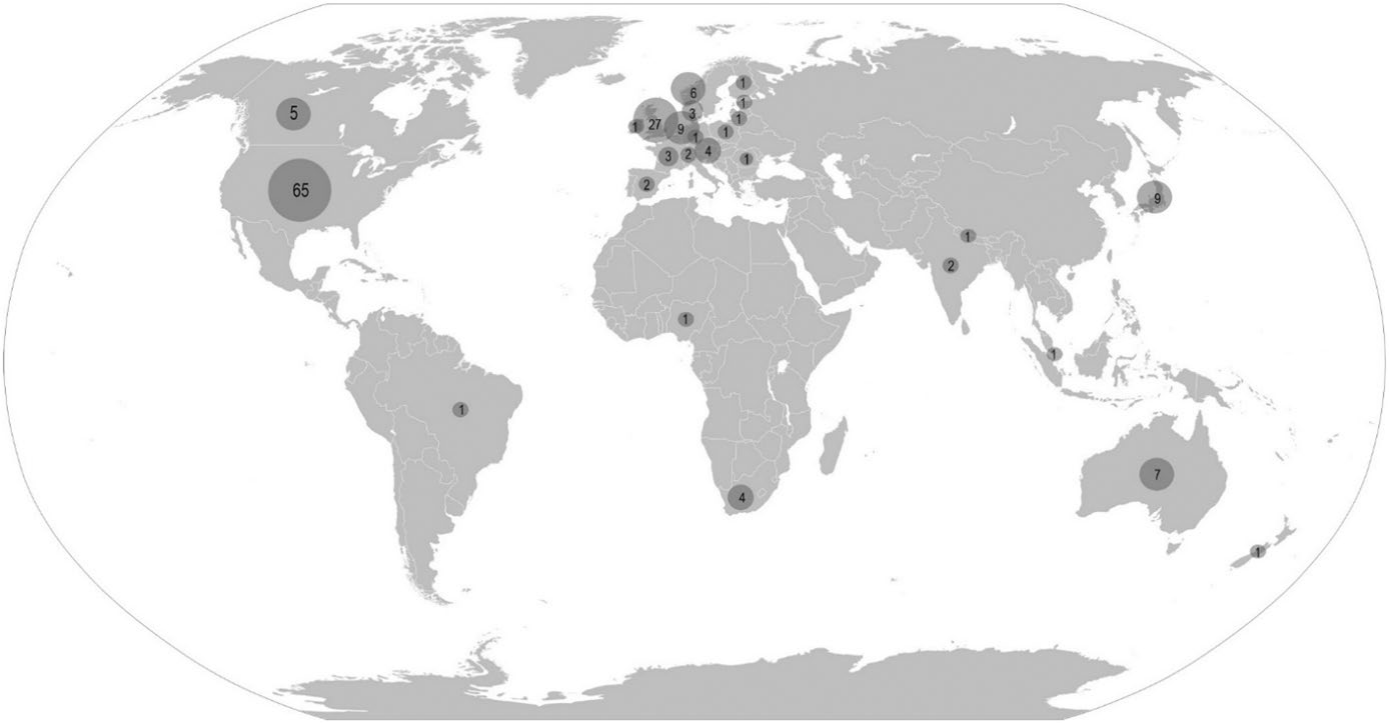
Origin of practices by country (without international practices; number of international practices n = 52). The United States of America (n = 65), United Kingdom (n = 27), Japan (n = 9), the Netherlands (n = 9), Australia (n = 7), Norway (n = 6), Canada (n = 5), Austria (n = 4), South Africa (n = 4), Denmark (n = 3), France (n = 3), India (n = 2), Spain (n = 2), Switzerland (n = 2), Brazil (n = 1), Estonia (n = 1), Finland (n = 1), Germany (n = 1), Ireland (n = 1), Lithuania (n = 1), Nepal (n = 1), New Zealand (n = 1), Nigeria (n = 1), Poland (n = 1), Romania (n = 1), Singapore (n = 1). There were 52 documents which were international and could not be located to a single country. Source for the geographical map: https://commons.wikimedia.org/wiki/File:BlankMap-World.svg (public domain)

The largest number of documents was related to practices from the USA (n = 65), followed by practices that were developed by international organisations or projects and not aimed at or developed by a specific country or countries, but instead could be applicable internationally (n = 50). Some examples of the practices that we mapped as international are Responsible Conduct in the Global Research Enterprise: A Policy Report by Inter Academy Council and the Inter Academy Partners (IAC and IAP 2012), World Health Organisation Guidelines for Good Clinical Practice for Trials on Pharmaceutical Products (WHO 1995) European Science Foundation Good scientific practice in research and scholarship (ESF 2000), and the Hong Kong Principles for Assessing Researchers: Fostering Research Integrity (Moher et al. 2019). Some documents contained the descriptions of practices related to more than one country, i.e. two or more counties were explicitly mentioned. In those cases, we included all the mentioned countries in the analysis. The origin of practices by country and the number of identified sources related to a particular country are presented in Fig. 2.

In terms of scientific fields, the majority of documents referred to RI issues that are not related to any specific field, i.e. research in general (n = 117), followed by documents that addressed RI in medical research (n = 78). We identified 10 documents for RI practices in social sciences, 10 for natural sciences (including engineering), and 4 related to RI practices in the field of humanities. Some documents referred to more than one scientific field, and in those cases, we counted each scientific field that was addressed. The most significant number of items included in the final analysis were practices that were more related to RPOs (n = 150). Although some practices related to RPOs were related to RFOs as well, we considered these practices to be primarily intended for RPOs since the guidance addressing the RFOs was only briefly mentioned. Guidance related equally to RPOs and RFOs was identified in 54 documents. Practices related to RFOs were identified in only 6 documents.

### Type of Guidance for RI Promotion.

Based on the distinction between the types of guidance on RI issues, we identified 11 types of practices. Among them, guidelines were most prevalent (n = 136). Other identified types of guidance were codes (n = 35), policies (n = 26), legal documents (n = 14), reports (n = 10), checklists (n = 9), statements (n = 6), declarations (n = 4), flowcharts (n = 2), white papers (n = 1), and standard operating procedures (n = 1). Some sources of practices referred to more than one type of guidance, and in these cases we counted and mapped each practice that was mentioned. For this reason, the numbers that are presented are higher than the number of documents included in the final analysis.

**Fig. 3:**
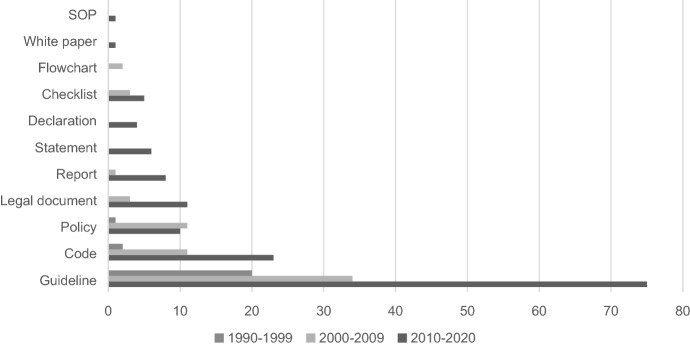
The number of practices in different time periods. The x-axis shows the number of practices, and the y-axis lists different types of practices. *SOP* standard operating procedure

We analysed the number of different types of guidance identified in this study over three time periods: 1990–1999, 2000–2009, and 2010–2019 (**Fig. ****3**). Most of the identified practices dated from 2010 onward and the guidelines were mostly represented throughout all the three time periods. For some practices (n = 11), we were not able to define the exact time when they were developed, hence we did not include them in this analysis.

### Target Group to Which Practices were Directed

RI practices addressed different individuals and organisations as target groups (Table 1). We grouped different individuals and organisations in the research chain that practices were aimed at into five primary categories: researchers, RPOs, RFOs, RE or RI bodies and other policymakers respectively. Most of the practices referred to more than one category.

**Table 1:** Individuals and organisations addressed in identified RI practices

Individuals and organisations by category and sub-category	No. of documents
Researchers (including research groups, students, mentors and supervisors, reviewers, whistle-blowers)	167
RPOs (including administrators)	111
Research integrity and research ethics bodies (REC, RIC, research councils, IRB, RIOs, RIAs, Ombudsman)	51
RFOs	41
Policymakers	41

*IRB* institutional review board; *REC* research ethics committee; *RI* research integrity; *RIA* research integrity advisor; *RIC* research integrity committee; *RIOs* research integrity offices/officers; *RFOs* research funding organisations; *RPOs* research performing organisations

### Research Processes and RI Topics Identified in the Documents

We first classified the documents according to the steps of the research process—planning, conducting, dissemination, evaluation—as well as according to RI violations and resolutions and RI promotion. These were then broadened by the list of RI-related topics that were mentioned in the analysed documents. For example, in the category of ‘RI violations and resolutions’ we put documents that addressed research misconduct investigations, sanctions and other, while in the category of ‘RI promotion’ we put documents related to the development and implementation of RI practices, implementation of RI training and establishment of RI bodies. Some topics were related to more than one research process. We analysed which of the extracted topics were related to RPOs, RFOs, and/or other policymakers. These practices reflected on the organisational procedures and measures that could be put into effect for individual researchers and for RI improvement in general. Classification by research processes and RI topics, together with the list of documents aimed at organisational level is available in Appendix 4 (Electronic Supplementary Material). The list of practices aimed at individual researchers only is presented in Appendix 5 (Electronic Supplementary Material).

### Principles Addressed in Guidance Documents

Some documents (n = 28), in addition to more specific guidance, contained fundamental guiding principles and values that researchers and organisations should follow. We matched these principles to those outlined in two major policy documents: the European Code of Conduct for Research Integrity (the ALLEA code) (2017) and the National Academies of Sciences, Engineering and Medicine (NASEM) book *Fostering Integrity in Research* (2017). The comparison of fundamental principles is available in Appendix 6 (Electronic Supplementary Material).

Regarding the type of documents in which the principles were addressed, the majority were codes (n = 13), followed by guidelines (n = 9), statements (n = 3), and policies (n = 4). The list and description of all extracted principles are available in Appendix 7 (Electronic Supplementary Material).

## Discussion

Our scoping review identified a number of available practices for the improvement of RI and research in general at RPOs and RFOs. Most of these practices were related to RPOs, in the form of guidelines, and addressed the RI topics related to the processes we categorised in our study as ‘RI violations and resolutions’, as well as ‘RI promotion’. The fact that only a small number of identified practices were related to RFOs shows the differences regarding RI in the context of different types of organisations. While the majority of identified RI practices were developed for research in general and could be applicable across different scientific fields, a small number of disciplinary-tailored guidance for fostering RI was identified in the natural sciences (including engineering), social sciences, and humanities. Besides practices that could be applicable across various disciplinary fields, this review showed that a substantial amount of RI practices were explicitly developed for medical sciences (including biomedicine).

While most of the practices were more related to RPOs, the gap in knowledge on RI guidance was noticed in the number of identified practices for RFOs. Some guidance documents, which were mapped as those related more to RPOs, briefly mentioned funders as important stakeholders in the research process. However, there were only a few examples of practices related solely to RFOs and their specific initiatives in fostering RI. In the context of RI, this can be problematic because RFOs, together with RPOs, play an important role in influencing researchers’ good or bad scientific behaviour (NASEM 2017). Although researchers build their career within RPOs and their behaviour is often influenced by organisational climate and policies, RFOs can impose additional safeguards if RPOs fail to promote and protect the integrity of their research. Usually, these measures by RFOs are aimed at RPOs rather than individual researchers although in some cases RFOs and researchers have a direct relationship (for example when setting out calls for funding, selecting certain projects to fund and monitoring funded projects). However, by demanding the establishment of RI promotion policies and procedures from RPOs, RFOs indirectly impact also the behaviour of individual researchers (Bouter 2018). Some of the important requests that RFOs may impose to RPOs for safeguarding RI may include a request for implementation of clear procedures for handling research misconduct or request for compliance with principles of open science and transparency in research publications (Bouter 2016).

Furthermore, the analysis of stakeholders at whom the documents were aimed showed that although a large number of practices addressed RPOs (organisational directors, managers and boards), most practices addressed individual researchers. A small number of guidance documents was directed for RI structures such as RI offices, committees or advisors. This could be because many organisations still do not have specific bodies appointed to deal with RI issues; instead, RI issues are handled by ethics committees (Marušić 2019). Additionally, research processes and RI topics analysis showed that efforts to establish RI bodies are emphasised as an important role of policymakers and organisational management, but mostly in newer documents dating after 2010.

The finding that most practices for RI promotion originated from the United States may be due to our methodology, which included the search of the United States (US) Office of Research Integrity (ORI) website and the publications of the US National Academies of Sciences, Engineering, and Medicine. A large number of identified guidance documents were also from the United Kingdom, which could be because our grey literature search was limited to documents written in English. Moreover, many documents were identified as ones that could be applied widely, i.e. internationally, regardless of the country-specific differences. These documents were mostly developed as efforts of collaborative projects and international organisations that deal with RI issues. To conclude on the usage of these documents, it would be necessary to additionally explore which documents were implemented across organisations in different countries. This, of course, excludes the international legislative documents, for example those of the European Union (EU), which are mandatory for EU member states.

Guidelines were the most common form of RI guidance identified in this study. However, there was considerable variability in the topics covered and the level of elaboration presented in different guidelines. Some guidelines were focused on a single RI issue or specific stakeholders and described the specific procedures in detail, for example on data management (Science Europe 2018) or how to respond to misconduct in research (MEXT 2014). Others presented various RI issues in a more general manner with the addition of specific recommendations and were aimed toward different stakeholders (NESH 2016, NASEM 2017). Only one of the guidance documents in this study was in the form of SOPs (n = 1). Although RPOs and RFOs probably have SOPs for different kinds of administrative issues, in this study we focused on the SOPs for RI, which perhaps not all research organisations have and our study suggests that SOPs focused on RI might be rare. Further, another reason may be that RPOs and RFOs do not publish their internal SOPs which may also include the SOPs related to RI issues. The approach offered by SOPs could be helpful for the initiatives supporting research organisations and researchers on their path to integrity (Bouter 2020). For example, SOPs could be developed for defining responsibilities or describing a procedure that should be performed in the same manner, such as uploading research results to a repository or the registration of research protocol. The same could be applicable for RI bodies when it comes to handling the cases of misconduct to ensure that the same procedure, from investigation to sanctioning, was followed in each case (Lerouge and Hol 2020).

The analysis of processes and RI topics for RPOs, RFOs, and other policymakers brought up several RI issues that were emphasised across identified practices as responsibilities of those at the organisational level. Most of these practices were related to the processes of ‘RI violations and resolutions’ and ‘RI promotion’. For the ‘RI violations and resolutions’ most documents were focused on RI topics related to describing processes of investigating and handling misconducts, as well as the importance on providing clear definitions of what constitutes research misconduct. For the ‘RI promotion’ most documents were focused on the development and implementation of RI policies and establishment of RI bodies. Providing RI training courses and education, as well as developing infrastructure for adequate data management were also mentioned in many documents as an important responsibility of research organisations. All this reflects the organisations’ valuable role in creating an environment and organisational culture in which researchers will be motivated to pertain to RI principles and rules in their work (Forsberg et al. 2018; Moher et al. 2019; Lerouge and Hol 2020).

The analysis of guiding principles showed that, although the naming of the principles was not consistent through all documents, the meaning of the principles in RI perspective was mostly the same. For example, the ALLEA code emphasises the principle of ‘reliability’ as employing a research methodology that will help enhance the quality of research, as well as to help ensure the trustworthiness of one’s work (ALLEA 2017). In NASEM, the same guidance regarding the validity of research was described under the principle of ‘accountability’ (NASEM 2017). However, ‘accountability’ is also used to demonstrate the responsibility of researchers toward research organisations and society (NASEM 2017) which corresponds to the principle of ‘accountability’ as described in the ALLEA code. The principles of ‘honesty’ in the ALLEA code, is defined as being honest and fair in every step of the research, valuing transparency in reporting research, as well as having an unbiased approach to the research tasks (ALLEA 2017). NASEM explicitly defines two other principles besides honesty—‘objectivity’ and ‘openness’—which emphasise avoiding biases and transferring the real results of research to the community. The principle of ‘respect’ by the ALLEA code is directed toward different parties involved in research, starting from other researchers and collaborators to the research participants and society. NASEM describes respect toward others involved in research by using the terms ‘stewardship’ and ‘fairness’. The ways of emphasising ‘respect’ were the most diverse regarding the terms used by different documents in comparison with other main principles. The variety of principles used across documents showed an overview of what values need to be taken into account when considering RI issues. However, general guidance might not be enough in judging research misbehaviours and principles can be used as a valuable starting point in creating more specific guidance documents.

### Study Strengths and Limitations

The main strength of this study is a comprehensive literature search that encompassed both peer-reviewed documents and grey literature from various sources and was performed according to a rigorous methodology that required documents to be screened by multiple researchers. This comprehensive search allowed us to create a library of documents containing RI practices that could be used by organisations and individual researchers in different scientific fields. It also helped us identify gaps in the currently existing practices for RPOs and RFOs and thus create opportunity for further development of RI practices and RI in general.

One of the possible limitations of our study could be that we may have missed important documents during the assessment of titles and abstracts, because the information provided therein was not sufficient for the inclusion in the analysis. Besides that, we were not able to perform the search of documents from every existing RPO and RFO and our grey literature search was limited to documents in the English language only. Therefore, we can assume that there are certainly more good practices that have not been included into this study. However, expanding our search to various RPOs’ and RFOs’ websites, as well as to include grey literature in languages other than English would raise a question of feasibility. Furthermore, the accessibility of guidance documents on RI may be low, as was shown for 18 universities from 10 European countries (Aubert Bonn et al. 2017), meaning that the search of individual organisations’ websites would not provide a comprehensive insight into the totality of the RI guidance at RPOs and RFOs. We were unable to retrieve five documents, but they dated from the nineties, so the guidance presented in them is potentially obsolete or has already been captured in contemporary documents. Since the aim of the study was to map the existing RI practices and gaps in the content of practices, we did not take into account whether there were interventions regarding the effectiveness of the identified practices.

## Conclusion

Although practices for RI promotion and initiatives to improve RPOs’ and RFOs’ effort in fostering RI exist it seems that more initiatives are needed for funders, RI bodies, and in certain disciplinary fields. As far as the form in which RI guidance is presented is concerned, it varies from general guidance outlining the principles and values that stakeholders should follow to more specific guidance for RI issues that are procedural in nature. When dealing with the latter, researchers and other stakeholders could find SOPs, checklists, and flowcharts to be a valuable resource of RI guidance. Through a systematic and thorough literature search, we collected a significant number of documents that RPOs and RFOs could use as guidance on RI issues or as inspiration for the development of new policies. Further research to determine factors, facilitators and barriers that may influence the implementation of RI practices could additionally help RPOs and RFOs in fostering RI.

## Supplementary Information

Below is the link to the electronic supplementary material.

Supplementary material 1 (DOCX 16 kb)

Supplementary material 2 (DOCX 14 kb)

Supplementary material 3 (DOCX 84 kb)

Supplementary material 4 (DOCX 43 kb)

Supplementary material 5 (DOCX 50 kb)

Supplementary material 6 (DOCX 23 kb)

Supplementary material 7 (DOCX 17 kb)

## Data Availability

The search strategies used to obtain documents for the analysis are available in Appendices 1 and 2. The list of all documents used in analysis and data extracted from the documents is available in Appendix 3. Research processes and RI topics identified across practices and list of documents in which RI topics were addressed toward organisations and policymakers are available in Appendix 4. List of documents for individual researchers, categorised by research processes and RI topics is available in Appendix 5. Comparison of fundamental principles from the ALLEA code NASEM – Fostering Integrity in Research book and matching principles found in other documents is available in Appendix 6. List of guiding principles extracted from documents is available in Appendix 7. All appendices are available as electronic supplementary material at the Open Science Framework (OSF). The data are available at the link below: https://osf.io/byw8s/?view_only=33369ee20c0a46cd9702f6ab8d6d1ad9.

## Abbreviations

ALLEA: All European Academies
CORDIS: Community Research and Development Information Service
ENRIO: European Network of Research Integrity Offices
EU: European Union
JBI: Joanna Briggs Institute
LERU: League of European Research Universities
MEXT: Ministry of Education, Culture, Sports, Science and Technology, Japan
MLE: Mutual Learning Exercises
NASEM: National Academies of Sciences, Engineering, and Medicine
NESH: The National Committee for Research Ethics in the Social Sciences and the Humanities, Norway
ORI: Office of Research Integrity (United States)
RE: Research Ethics
RI: Research Integrity
RFO: Research Funding Organisation
RPO: Research Performing Organisation
SOP: Standard Operating Procedures
US: United States
WCRI: World Conferences on Research Integrity
WOS: Web of Science
