# Supplementary material for: Practices for Research Integrity Promotion in Research Performing Organisations and Research Funding Organisations: A Scoping Review
Source: Sci Eng Ethics. 2021 Jan 27;27(1):4. doi: 10.1007/s11948-021-00281-1 (PMC7840650; doi:10.1007/s11948-021-00281-1)
Supplement: Supplementary file 1 — Supplementary material 1 (DOCX 16 kb) [file 11948_2021_281_MOESM1_ESM.docx]

**Appendix 1 Search strategies for bibliographic databases**

**a) Scopus**

(TITLE-ABS-KEY(research W/3 (integrity OR ethics OR conduct OR misconduct OR malpractice OR manipulation OR fraud* OR honest*))) OR (TITLE-ABS-KEY((scientific OR academic) W/3 (fraud OR ethics OR integrity OR misconduct OR honesty OR dishonesty))) OR (TITLE-ABS-KEY((researcher* OR scientist*) W/3 (integrity OR honest*))) OR (TITLE-ABS-KEY((publication* or publishing) W/3 (ethics OR plagiari* OR falsif*)) OR (TITLE-ABS-KEY((author* OR contribut*) W/3 (undeserv* OR ghost OR guest OR gift*)))) AND ((TITLE-ABS-KEY(code W/3 (ethic* or conduct)) OR (TITLE-ABS-KEY(educat* OR teach* OR train* OR motivat* OR instruct* OR interven* OR promot* OR supervis* OR mentor*)) OR (TITLE-ABS-KEY(course* OR seminar* OR workshop*)) OR (TITLE-ABS-KEY((program* OR plan* OR policy OR rule* OR procedure* OR standard* OR code*) W/3 (formulat* OR develop* OR improve* OR expand*))) OR (TITLE-ABS-KEY(quality control))) AND (TITLE-ABS-KEY((ethics or research or grant or grants) W/3 (committee or committees or commission or commissions))) OR (TITLE-ABS-KEY(research W/3 (organisation* OR organization*)) OR (TITLE-ABS-KEY(universit$ or college or colleges)) OR (TITLE-ABS-KEY (universit* AND (faculty or faculties or school or schools or department or departments or laboratory or laboratories or lab or institut or institute or institutes))) OR (TITLE-ABS-KEY(academic or academia or higher education*))))

**b) Web of Science**

# 20 #19 AND #13 AND #6

# 19 #18 OR #17 OR #16 OR #15 OR #14

# 18 TS=(academic OR academia OR higher education*)

# 17 TS=(universit* AND (faculty OR faculties OR school OR schools OR department OR departments OR laboratory OR laboratories OR lab OR institut OR institute OR institutes))

# 16 TS=(universit* OR college OR colleges)

# 15 TS=(research NEAR/3 (organisation* OR organization*))

# 14 TS=((ethics OR research OR grant OR grants) NEAR/3 (committee OR committees OR commission OR commissions))

# 13 #12 OR #11 OR #10 OR #9 OR #8 OR #7

# 12 TS=(quality NEAR/3 control*)

# 11 TS=((program* OR plan* OR policy OR rule* OR procedure* OR standard* OR code*) NEAR/3 (formulat* OR develop* OR improve* OR expand*))

# 10 TS=(course* OR seminar* OR workshop*)

# 9 TS=(educat* OR teach* OR train* OR motivat* OR instruct* OR interven* OR promot* OR supervis* OR mentor*)

# 8 TS=(code NEAR/3 (ethic* or conduct))

# 7 TS=(guideline*)

# 6 #5 OR #4 OR #3 OR #2 OR #1

# 5 TS=((author* OR contribut*) NEAR/3 (undeserv* OR ghost OR guest OR gift*))

# 4 TS=((publication* OR publishing) NEAR/3 (ethics OR plagiari* OR falsif*))

# 3 TS=((researcher* OR scientist*) NEAR/3 (integrity OR honest*))

# 2 TS=((scientific OR academic) NEAR/3 (fraud OR ethics OR integrity OR misconduct OR honesty OR dishonesty))

# 1 TS=(research NEAR/3 (integrity OR ethics OR conduct OR misconduct OR malpractice OR manipulation OR fraud* OR honest*))

**c) Medline**

1 Scientific Misconduct/ (5023)

2 Fraud/ (7036)

3 exp Ethics, Research/ (7574)

4 (research adj3 (integrity or ethics or conduct or misconduct or malpractice or manipulation or misleading or mispresent$ or bias$ or fraud$ or honest$ or reliab?l$ or fair$ or impartial$ or selective$)).tw. (15995)

5 ((scientific or academic) adj3 (fraud or ethics or integrity or misconduct or malpractice or manipulation or honesty or dishonesty)).tw. (2418)

6 ((researcher$ or scientist$) adj3 (integrity or honest$)).tw. (92)

7 Plagiarism/ (1214)

8 (plagiari$ or falsif$).tw. (3121)

9 Publication Bias/ (4693)

10 Duplicate Publication as Topic/ (757)

11 Retraction of Publication as Topic/ (594)

12 Peer Review, Research/ (6325)

13 (data adj3 (interpretat$ or inaccura$ or inadequa$ or deceptive or deceit or bias$ or impartial or manipulat$ or misus$ or misleading or mispresent$ or mistreat$ or selective or suppress$ or fabricat$ or fraud$ or falsif$ or false)).tw. (27201)

14 Research Report/ (2769)

15 (report$ adj3 (selective or deceptive or deceit or misleading or inadequate or independent)).tw. (6958)

16 (research adj3 (underreport$ or under-report$)).tw. (43)

17 ((publication$ or publishing) adj3 ethics).tw. (485)

18 (bias adj3 (publication$ or publishing or analys#s or design)).tw. (13061)

19 (publication$ adj3 (rendundant or duplicate or multiple or salami or undeserving)).tw. (875)

20 (inaccura$ adj3 citation$).tw. (17)

21 Authorship/ (5535)

22 ((author$ or contribut$) adj3 (undeserv$ or ghost or guest or gift$)).tw. (258)

23 Conflict of Interest/ (9252)

24 (interest adj3 (conflict or competing)).tw. (4281)

25 or/1-24 (108903)

26 exp guideline/ (31503)

27 guideline$.tw. (304028)

28 exp "Codes of Ethics"/ (5164)

29 (code adj3 (ethic$ or conduct)).tw. (2457)

30 exp Education, Professional/ (282429)

31 exp Teaching/ (80510)

32 exp Curriculum/ (79237)

33 Mentors/ (9918)

34 (educat$ or teach$ or train$ or motivat$ or instruct$ or interven$ or promot$ or supervis$ or mentor$).tw. (2738959)

35 (course$ or seminar$ or workshop$).tw. (612665)

36 Policy/ (2054)

37 exp Policy Making/ (24148)

38 Program Development/ (27358)

39 ((program$ or plan$ or policy or rule$ or procedure$ or standard$ or code$) adj3 (formulat$ or develop$ or improve$ or expand$)).tw. (181855)

40 Quality Control/ (46654)

41 (quality adj3 control$).tw. (50594)

42 or/26-41 (3811000)

43 exp Ethics Committees/ (9027)

44 ((ethics or research or grant or grants) adj3 (committee or committees or commission or commissions)).tw. (13582)

45 (research adj3 organi#ation$).tw. (8560)

46 Universities/ (36926)

47 (universit$ or college or colleges).tw. (416213)

48 (universit$ and (faculty or faculties or school or schools or department or departments or laboratory or laboratories or lab or institut or institute or institutes)).tw. (106436)

49 (academic or academia or higher education$).tw. (129189)

50 or/43-49 (560208)

51 25 and 42 and 50 (6001)

**d) PsychINFO**

1 fraud/ (809)

2 professional ethics/ (18329)

3 (research adj3 (integrity or ethics or conduct or misconduct or malpractice or manipulation or misleading or mispresent$ or bias$ or fraud$ or honest$ or reliab?l$ or fair$ or impartial$ or selective$)).tw. (11366)

4 ((scientific or academic) adj3 (fraud or ethics or integrity or misconduct or malpractice or manipulation or honesty or dishonesty)).tw. (1345)

5 ((researcher$ or scientist$) adj3 (integrity or honest$)).tw. (77)

6 plagiarism/ (240)

7 (plagiari$ or falsif$).tw. (2533)

8 peer evaluation/ (2761)

9 peer review$.tw. (7868)

10 (data adj3 (interpretat$ or inaccura$ or inadequa$ or deceptive or deceit or bias$ or impartial or manipulat$ or misus$ or misleading or mispresent$ or mistreat$ or selective or suppress$ or fabricat$ or fraud$ or falsif$ or false)).tw. (7597)

11 (report$ adj3 (selective or deceptive or deceit or misleading or inadequate or independent)).tw. (1707)

12 (research adj3 (underreport$ or under-report$)).tw. (17)

13 ((publication$ or publishing) adj3 ethics).tw. (183)

14 (bias adj3 (publication$ or publishing or analys#s or design)).tw. (2638)

15 (publication$ adj3 (rendundant or duplicate or multiple or salami or undeserving)).tw. (150)

16 (inaccura$ adj3 citation$).tw. (13)

17 ((author$ or contribut$) adj3 (undeserv$ or ghost or guest or gift$)).tw. (452)

18 Conflict of Interest/ (564)

19 (interest adj3 (conflict or competing)).tw. (1343)

20 or/1-19 (54985)

21 guideline$.tw. (58798)

22 (code adj3 (ethic$ or conduct)).tw. (2909)

23 education/ (32620)

24 teaching/ (42029)

25 curriculum/ (25054)

26 mentor/ (5836)

27 (educat$ or teach$ or train$ or motivat$ or instruct$ or interven$ or promot$ or supervis$ or mentor$).tw. (1395167)

28 (course$ or seminar$ or workshop$).tw. (200665)

29 exp policy making/ (68897)

30 exp program development/ (8798)

31 ((program$ or plan$ or policy or rule$ or procedure$ or standard$ or code$) adj3 (formulat$ or develop$ or improve$ or expand$)).tw. (67869)

32 quality control/ (1434)

33 (quality adj3 control$).tw. (3335)

34 or/21-33 (1597178)

35 ((ethics or research or grant or grants) adj3 (committee or committees or commission or commissions)).tw. (2402)

36 (research adj3 organi#ation$).tw. (8713)

37 colleges/ (13109)

38 (universit$ or college or colleges).tw. (327580)

39 (universit$ and (faculty or faculties or school or schools or department or departments or laboratory or laboratories or lab or institut or institute or institutes)).tw. (45016)

40 (academic or academia or higher education$).tw. (156810)

41 or/35-40 (451152)

42 20 and 34 and 41 (5330)
