# Supplementary material for: Practices for Research Integrity Promotion in Research Performing Organisations and Research Funding Organisations: A Scoping Review
Source: Sci Eng Ethics. 2021 Jan 27;27(1):4. doi: 10.1007/s11948-021-00281-1 (PMC7840650; doi:10.1007/s11948-021-00281-1)
Supplement: Supplementary file 2 — Supplementary material 2 (DOCX 14 kb) [file 11948_2021_281_MOESM2_ESM.docx]

**Appendix 2 Search of grey literature sources**

**a) Open Grey database**

Documents were found using the terms ‘research ethics’ and ‘research integrity’. The process of screening included the screening of titles and abstracts, followed by full-text analysis.

**b) CORDIS database**

Relevant projects were identified using the term ‘research integrity’. Projects documents (deliverables, publications, etc.) were screened for the identification of documents related to research integrity (RI) practices.

**c) World Conferences on Research Integrity (WCRI)**

The search was performed on the web pages of the World Conferences on Research Integrity (WCRI). The aim was to identify suitable conference material, abstracts, PowerPoint presentations from lectures and workshops related to RI practices.

**d) United States Office of Research Integrity (ORI)**

The search was performed on the ORI web pages. The search aimed to identify publications related to RI and responsible conduct of research (RCR) practices.

**e) European Network of Research Integrity Offices (ENRIO)**

The search was performed on ENRIO web pages. The search aimed to identify publications containing RI practices.

**f) The National Academies of Sciences, Engineering, and Medicine (NASEM)**

The search was performed on the NASEM web pages to identify publication related to RI and RCR.

**g) Science Europe**

The search was performed on the web pages of Science Europe to identify publications related to RI.

**h) Mutual Learning Exercise on Research Integrity**

The search was performed on the web pages of the European Commission to identify reports published by the Mutual Learning Exercise on Research Integrity working group.

**i) League of European Research Universities (LERU)**

The search included the screening of the publication *Towards a Research Integrity Culture at Universities: From Recommendations to Implementation* (Lerouge and Hol 2020).
