# Supplementary material for: Practices for Research Integrity Promotion in Research Performing Organisations and Research Funding Organisations: A Scoping Review
Source: Sci Eng Ethics. 2021 Jan 27;27(1):4. doi: 10.1007/s11948-021-00281-1 (PMC7840650; doi:10.1007/s11948-021-00281-1)
Supplement: Supplementary file 4 — Supplementary material 4 (DOCX 43 kb) [file 11948_2021_281_MOESM4_ESM.docx]

**Appendix 4** **Research processes and RI topics identified across practices and list of documents in which RI topics were addressed toward RPOs, RFOs, and other policymakers**

| **Research process** | **RI topics** |
| --- | --- |
| **Research planning** | **Authorship (including publication plan)** (Graf et al. 2009; Morris 2010; NASEM 2017; CSE 2018; Wellcome Trust 2018)  **Consideration of ethical issues (including risk-benefit assessment)** (WHO 2005; KNAW 2008; UKRIO 2009; Resnik and Shamoo 2011; Euro Scientist 2017; WEF 2018; Wellcome Trust 2018)  **Research methodology** (NESH 2016; NASEM 2017; Wellcome Trust 2018; Parder and Juurik 2019; Lerouge and Hol 2020) |
| **Research conducting** | **Authorship** (NASEM 2017; NHMRC 2019a; Lerouge and Hol, 2020)  **Collaboration** (UKRIO 2009; DFG 2013; Montreal Statement 2013; Danish Ministry of Higher Education and Science 2014; IUA 2014; Duggins Peloso et al. 2015; Boeheme et al. 2016; ALLEA 2017; Euro Scientist 2017; NASEM 2017; University of Tartu 2017; SAMRC 2018; Wellcome Trust 2018; Marušić 2019a; Parder and Juurik 2019)  **Conflict of interest** (National Academy of Sciences, National Academy of Engineering, and Institute of Medicine 1993; Lo and Field 2009; UKRIO 2009; Danish Ministry of Higher Education and Science 2014; IUA 2014; Kyoto University 2014; Dade et al. 2015; NENT 2016; NESH 2016; NASEM 2017; University of Tartu 2017; CSE 2018; Toom and Miller 2018; ENERI, ENRIO and OeAWI 2019; Marušić 2019b; NHMRC 2019b; University of Oxford 2019a; USQ 2019)  **Data management** (National Academy of Sciences, National Academy of Engineering, and Institute of Medicine 1993; EMA 2002; WHO 2005; Danish Committees on Scientific Dishonesty 2009; National Academy of Sciences, National Academy of Engineering, and Institute of Medicine 2009a; UKRIO 2009; DFG 2013; KNAW 2013; Danish Ministry of Higher Education and Science 2014; IUA 2014; QUB 2014; Hiney 2015; Sallans and Patterson 2015; ALLEA 2017; Aoki et al. 2017; NASEM 2017; University of Tartu 2017; NASEM 2018a; NASEM 2019; Netherlands Code of Conduct for Research Integrity 2018; NHMRC 2018a; SAMRC 2018; Science Europe 2018a; Science Europe 2018b; Wellcome Trust 2018; NHMRC 2019c; Universities UK 2019; Lerouge and Hol 2020; Science Europe 2020; University of Oxford)  **Data protection (privacy and confidentiality)** (WHO 2005; UKRIO 2009; Danish Ministry of Higher Education and Science 2014; PhRMA 2014; Epstein and Lascher 2015; NESH 2016; NASEM 2017; University of Tartu 2017; Araki et al. 2018, Eckstein et al. 2018; Penders et al. 2018; University of Oxford 2018b; NHMRC 2019c; University of Oxford 2019b)  **Intellectual property and data ownership** (Bertha 1996; EC 2005; Graf et al. 2009; UKRIO 2009; Danish Ministry of Higher Education and Science 2014; QUB 2014; University of Tartu 2017; CSE 2018; Parder and Juurik 2019)  **Mentorship/supervision** (National Academy of Sciences, National Academy of Engineering, and Institute of Medicine 1992; National Academy of Sciences, National Academy of Engineering, and Institute of Medicine 1993; EC 2005; UKRIO 2009; DFG 2013; Danish Ministry of Higher Education and Science 2014; NASEM 2017; University of Tartu 2017; Forsberg et al. 2018; NASEM 2018b; Netherlands Code of Conduct for Research Integrity 2018; NHMRC 2018a; SAMRC 2018; WEF 2018; Wellcome Trust 2018; Lerouge and Hol 2020; ENERI) |
| **Research dissemination** | **Authorship** (Graf et al. 2009; NASEM 2017; Lerouge and Hol 2020)  **Open science** (Science Europe 2013; Hiney 2015; Science Europe 2015; NENT 2016; NESH 2016; ALLEA 2017; NASEM 2017; University of Tartu 2017; Breit et al. 2018; Forsberg et al. 2018; NASEM 2018a; Science Europe 2018b; WEF 2018; Wellcome Trust 2018; Marušić 2019a; NASEM 2019; Parder and Juurik 2019; Lerouge and Hol 2020; Transparify)  **Reporting research** (Royal College of Physicians 2007; PhRMA 2014; NENT 2016; Euro Scientist 2017; WEF 2018; NASEM 2019; Lerouge and Hol 2020) |
| **Research evaluation** | **Audit** (EMA 2002; WHO 2005; Shimokai et al. 2007; UKRIO 2009; Epstein and Lascher 2015; Schaller-Demers 2015; Science Europe 2017)  **Ethical assessment** (Moodie and Marshall 1992; United Kingdom Health Ministers 1995; EFGCP 1997; Fagot-Largeault 2000; McIntosh et al. 2000; Eckstein 2003; WHO 2005; NHREC 2007; Royal College of Physicians 2007; Cleaton-Jones and Wassenaar 2010; NHRC 2011; Nys 2012; Danish Ministry of Higher Education and Science 2014; Epstein and Lascher 2015; Van Andel 2015; Piasecki et al. 2016; Netherlands Code of Conduct for Research Integrity 2018; NHMRC 2018b; SAMRC 2018; Toom and Miller 2018; USQ 2018; WMA 2018; NHMRC 2019d; OHRP 2019; Universities UK 2019; University of Oxford 2019b, ENERI; HHS)  **Evaluation of projects** (Institute of Medicine and National Research Council 2002; DORA 2012; Marušić 2019a)  **Peer review** (National Academy of Sciences, National Academy of Engineering, and Institute of Medicine 1993; Institute of Medicine and National Research Council 2002; UKRIO 2009; DFG 2013; NASEM 2017; Science Europe 2017; SAMRC 2018)  **Quality control** (WHO 1995; EMA 2002; Institute of Medicine and National Research Council 2002; PhRMA 2014; Epstein and Lascher 2015; Breit and Forsberg 2018; Forsberg et al. 2018)  **Research metrics** (National Academy of Sciences, National Academy of Engineering, and Institute of Medicine 1992; National Academy of Sciences, National Academy of Engineering, and Institute of Medicine 1993; Hicks et al. 2015; NASEM 2018b; Penders et al. 2018; WEF 2018; Fanelli 2019a; Moher et al. 2019)  **Research monitoring** (EMA 2002; Institute of Medicine and National Research Council, 2002; PhRMA 2014; NASEM 2017; NHMRC 2018b; Parder and Juurik 2019) |
| **RI violations and resolutions** | **Data protection in investigations** (EPA 2003; IUA 2014; MEXT 2014; NASEM 2017; Science Europe 2017; University of Oxford 2018a; Marušić 2019b; Universities UK 2019)  **Detrimental/questionable/poor research practices definitions** (National Academy of Sciences, National Academy of Engineering, and Institute of Medicine 1992; National Academy of Sciences, National Academy of Engineering, and Institute of Medicine 1993; National Academy of Sciences, National Academy of Engineering, and Institute of Medicine 2009b; KNAW 2013; IUA 2014; Antes 2015; Hiney 2015; OeAWI 2015; Schaller-Demers 2015; ALLEA 2017; NASEM 2017; ENERI, ENRIO and OeAWI 2019; Marušić 2019b)  **Fabrication, falsification, plagiarism and self-plagiarism definitions** (National Academy of Sciences, National Academy of Engineering, and Institute of Medicine 1992; National Academy of Sciences, National Academy of Engineering, and Institute of Medicine 1993; NSF 2002; EPA 2003; HHS 2005; Harvard Medical School 2005; OECD 2007; National Academy of Sciences, National Academy of Engineering, and Institute of Medicine 2009b; UKRIO 2009; KNAW 2013; IUA 2014; Hiney 2015; Kyoto University 2015; OeAWI 2015; Schaller-Demers 2015; Boeheme et al. 2016; ALLEA 2017; Dwivedi and Tripathi 2017; NASEM 2017; Science Europe 2017; Netherlands Code of Conduct for Research Integrity 2018; University of Oxford 2018a; ENERI, ENRIO and OeAWI 2019; Kyoto University 2019; Marušić 2019b; Universities UK 2019)  **Handling research misconduct (complaints and investigations)** (National Academy of Sciences, National Academy of Engineering, and Institute of Medicine 1992; National Academy of Sciences, National Academy of Engineering, and Institute of Medicine 1993; ORI 1995; ORI 1998; ESF 2000; Evans 2000; OSTP 2000; NSF 2002; EPA 2003; EC 2005; Harvard Medical School 2005; HHS 2005; SCJ 2006; OECD 2007; NTU 2008; UKRIO 2008; OECD 2009; UKRIO 2009; ESF 2011; Resnik and Shamoo 2011; TENK 2012; Wager and Kleinert 2012; DFG 2013; GRC 2013; Danish Ministry of Higher Education and Science 2014; IUA 2014; MEXT 2014; Boyd 2015; CSIC 2015; Kyoto University 2015; Boeheme et al. 2016; Israel and Drenth 2016; NESH 2016; Secretariat on Responsible Conduct of Research 2016; ALLEA 2017; Dwivedi and Tripathi 2017; NASEM 2017; Science Europe 2017; University of Tartu 2017; Breit et al. 2018; CSE 2018; Forsberg et al. 2018; Netherlands Code of Conduct for Research Integrity 2018; Toom and Miller 2018; University of Oxford 2018a; Wellcome Trust 2018; ENERI, ENRIO and OeAWI 2019; Kyoto University 2019; Marušić 2019b; NHMRC 2019c; Universities UK 2019; Lerouge and Hol 2020; ENERI)  **Sanctions** (NSF 2002; EC 2005; HHS 2005; SCJ 2006; NTU 2008; Danish Ministry of Higher Education and Science 2014; IUA 2014; MEXT 2014; Boyd 2015; Schaller-Demers 2015; Boeheme et al. 2016; Science Europe 2017; Netherlands Code of Conduct for Research Integrity 2018; NHMRC 2018a; SAMRC 2018; ENERI, ENRIO and OeAWI 2019; Kyoto University 2019; Marušić 2019a; Marušić 2019b; Parder and Juurik 2019)  **Whistle-blowers protection** (ORI 1995; OSTP 2000; EPA 2003; KNAW 2008; NTU 2008; DFG 2013; NENT 2016; NASEM 2017; Science Europe 2017; Breit et al. 2018; Forsberg et al. 2018; NHMRC 2018b; ENERI; Marušić 2019b; Parder and Juurik 2019; Universities UK 2019) |
| **RI promotion** | **Development and implementation of RI practices** (National Academy of Sciences, National Academy of Engineering, and Institute of Medicine 1992; National Academy of Sciences, National Academy of Engineering, and Institute of Medicine 1993; ESF 2000; Institute of Medicine and National Research Council 2002; OECD 2007; National Academy of Sciences, National Academy of Engineering, and Institute of Medicine 2009b; UKRIO 2009; ESF 2011; IAC and IAP 2012; Wager and Kleinert 2012; DFG 2013; GRC 2013; Danish Ministry of Higher Education and Science 2014; MEXT 2014; QUB 2014; Boyd 2015; CSIC 2015; OeAWI 2015; Schaller-Demers 2015; Boeheme et al. 2016; NENT 2016; NESH 2016; Secretariat on Responsible Conduct of Research 2016; ALLEA 2017; NASEM 2017; Science Europe 2017; Breit et al. 2018; Forsberg et al. 2018; NASEM 2018b; NHMRC 2018a; SAMRC 2018; Wellcome Trust 2018; ENERI, ENRIO and OeAWI 2019; ENERI; Kyoto University 2019; Marušić 2019a; Marušić 2019b; NHMRC 2019c; Universities UK 2019; Lerouge and Hol 2020; Science Europe 2020)  **Establishment and work of RI/RE bodies** (National Academy of Sciences, National Academy of Engineering, and Institute of Medicine 1992; ESF 2000; NHREC 2007; OECD 2007; Royal College of Physicians 2007; NTU 2008; UKRIO 2008; ESF 2011; Nys 2012; Wager and Kleinert 2012; DFG 2013; Danish Ministry of Higher Education and Science 2014; IUA 2014; Medical University of Graz 2014; MEXT 2014; Boyd 2015; CSIC 2015; Hiney 2015; Schaller-Demers 2015; Dwivedi and Tripathi 2017; NASEM 2017; Science Europe 2017; SATORI 2017; Breit and Forsberg 2018; Forsberg et al. 2018; NHMRC 2018a; NHMRC 2018b; Penders et al. 2018; ENERI, ENRIO and OeAWI 2019; Marušić 2019b; Universities UK 2019; Lerouge and Hol 2020; ENERI)  **Incentives** (National Academy of Sciences, National Academy of Engineering, and Institute of Medicine 1992; DORA 2012; NENT 2016; ALLEA 2017; Science Europe 2017; Breit and Forsberg 2018; Forsberg et al. 2018; NASEM 2018b; Wellcome Trust 2018; Fanelli 2019a; Lerouge and Hol 2020;)  **Research culture** (National Academy of Sciences, National Academy of Engineering, and Institute of Medicine 1992; National Academy of Sciences, National Academy of Engineering, and Institute of Medicine 1993; CEHAT 2000; EC 2005; OECD 2007; National Academy of Sciences, National Academy of Engineering, and Institute of Medicine 2009b; ESF 2011; Resnik and Shamoo 2011; IAC and IAP 2012; Danish Ministry of Higher Education and Science 2014; QUB 2014; CSIC 2015; Hiney 2015; Boeheme et al. 2016; NESH 2016; ALLEA 2017; NASEM 2017; Secretariat on Responsible Conduct of Research 2016; University of Tartu 2017; Breit et al. 2018; Forsberg et al. 2018; Netherlands Code of Conduct for Research Integrity 2018; Toom and Miller 2018; WEF 2018; Wellcome Trust 2018; Parder and Juurik 2019; Universities UK 2019; Lerouge and Hol 2020)  **RI training and education** (National Academy of Sciences, National Academy of Engineering, and Institute of Medicine 1992; National Academy of Sciences, National Academy of Engineering, and Institute of Medicine 1993; CEHAT 2000; Institute of Medicine and National Research Council 2002; EC 2005; SCJ 2006; OECD 2007; Royal College of Physicians 2007; NTU 2008; UKRIO 2009; ESF 2011; NIH 2011; IAC and IAP 2012; GRC 2013; KNAW 2013; NAE 2013; Danish Ministry of Higher Education and Science 2014; ICSU 2014; IUA 2014; MEXT 2014; QUB 2014; Antes 2015; Boyd 2015; CSIC 2015; Hausbeck Korgan 2015; Hendrickson 2015; Hiney 2015; Kyoto University 2015; Schaller-Demers 2015; Boeheme et al. 2016; Foeger and Zimmerman 2016; Israel and Drenth 2016; NESH 2016; Secretariat on Responsible Conduct of Research 2016; ALLEA 2017; NASEM 2017; Science Europe 2017; University of Tartu 2017; Breit et al. 2018; Forsberg et al. 2018; NASEM 2018b; Netherlands Code of Conduct for Research Integrity 2018; NHMRC 2018a; Toom and Miller 2018; WEF 2018; Wellcome Trust 2018; Fanelli 2019b; Kyoto University 2019; NASEM 2019; NHMRC 2019c; NHMRC 2019d; Parder and Juurik 2019; Universities UK 2019; Lerouge and Hol 2020; Science Europe 2020; ENERI) |

ALLEA – All European Academies; CEHAT – Centre for Enquiry into Health and Allied Themes; CSE – Council of Science Editors; DFG – Deutsche Forschungsgemeinschaft.; DORA – Declaration on Research Assessment; EC – European Commission; EFGCP – European Forum for Good Clinical Practice; EMA – European Medicines Agency; ENERI – European Network of Research Ethics and Research Integrity; ENRIO – European Network of Research Integrity Offices; EPA – Environmental Protection Agency; ESF – European Science Foundation; GRC – Global Research Council; HHS – Department of Health and Human Services (United States); IAC – Inter Academy Council; IAP – Inter Academy Partners; ICSU – International Council for Science; IUA – Irish Universities Association; KNAW – Royal Netherlands Academy of Arts and Sciences; MEXT – Ministry of Education, Culture, Sports, Science and Technology (Japan); NAE – National Academy of Engineering (United States); NASEM – National Academies of Sciences, Engineering, and Medicine (United States); NENT – National Committee for Research Ethics in Science and Technology (Norway); NESH – National Committee for Research Ethics in the Social Sciences and the Humanities (Norway); NHMRC – National Health and Medical Research Council (Australia); NHRC – Nepal Health Research Council; NHREC – National Health Research Ethics Committee (Nigeria); NIH – National Institutes of Health; NSF – National Science Foundation (United States); NTU – Nanyang Technological University; OeAWI – Austrian Agency for Research Integrity; OECD – Organisation for Economic Co-operation and Development; OHRP – Office for Human Research Protections (United States); ORI – Office of Research Integrity (United States); OSTP – Office of Science and Technology Policy (United States); PhRMA – Pharmaceutical Research and Manufacturers of America; QUB – Queen's University Belfast; RI – Research Integrity; SAMRC – South African Medical Research Council; SATORI – Stakeholders Acting Together On the ethical impact assessment of Research and Innovation; SCJ – Science Council of Japan; TENK – Finnish National Board on Research Integrity; UK – United Kingdom; UKRIO – United Kingdom Research Integrity Office; US – United States; USQ – University of Southern Queensland; WEF – World Economic Forum; WHO – World Health Organization

**References**

All European Academies (ALLEA). (2017). European Code of Conduct for Research Integrity. <https://allea.org/code-of-conduct/>. Accessed 17 June 2020.

Antes, A. L. (2015). Part II: Research Integrity: Navigating the Grey Areas of Scientific Work: Questionable Research Practices and Training in the Responsible Conduct of Research. In: A. Dade, O. Lori, & S. M. DiBella (Eds.), *Implementing a Comprehensive Research Compliance Program: A Handbook for Research Officers* (pp. 145–180). Charlotte, NC: Information Age Publishing, Inc.

Aoki, T., Kajita, S., Akasaka, H., & Takeda, H. (July 9-13, 2017). Development and Deployment of Research Data Preservation Policy at a Japanese Research University in 2016. 6th IIAI International Congress on Advanced Applied Informatics (IIAI-AAI), Hamamatsu, Japan.

Araki, K., Masuzawa, Y., Takahashi, Y., & Nakayama, T. (2018). [The Japanese legal system and the applicability of laws and regulations on private information protection and research ethics relating to medical research]. *Japanese Journal of Public Health*, 65(12), 730–743. <https://europepmc.org/article/med/30587680>. Accessed 15 June 2020.

Austrian Agency for Research Integrity (OeAWI). (2015). Guidelines for good scientific practice. <https://oeawi.at/wp-content/uploads/2018/09/OeAWI_Brosch%C3%BCre_Web_2019.pdf>. Accessed 15 June 2020.

Bertha, S. L. (1996). Academic research: policies and practice. *Journal of Ethnopharmacology*, 51, 59-73.

Boeheme, O., Foeger, N., Hiney, M., Peatfield, T., & Petiet, F. (2016). Research Integrity Practices in Science Europe Member Organisations. Science Europe: Science Europe Working Group on Research Integrity. <https://www.scienceeurope.org/our-resources/research-integrity-practices-in-science-europe-member-organisations/>. Accessed 15 June 2020.

Boyd, E. A. (2015). Part II: Research Integrity: Research Misconduct Programs and Role of the Research Integrity Officer. In: A. Dade, O. Lori, & S. M. DiBella (Eds.), *Implementing a Comprehensive Research Compliance Program: A Handbook for Research Officers* (pp. 101–122). Charlotte, NC: Information Age Publishing, Inc.

Breit, E., & Forsberg, E. (2018). Promoting Integrity as an Integral Dimension of Excellence in Research (PRINTEGER) project. Tools for research leaders and managers: addressing and stimulating integrity in research organisations.

https://printeger.eu/wp-content/uploads/2019/01/D5_2-Tools-for-research-leaders-and-managers.pdf. Accessed 17 June 2020.

Breit, E., Forsberg, E., & Vie, K. (2018). Promoting Integrity as an Integral Dimension of Excellence in Research (PRINTEGER) project. Managing research integrity: An assessment of best practices from the organisational literature.

<https://printeger.eu/wp-content/uploads/2018/06/3.9-organisational-best-practices.pdf>. Accessed 17 June 2020.

Centre for Enquiry into Health and Allied Themes (CEHAT). (2000). National Committee for Ethics in Social Science Research in Health (NCESSRH): Ethical Guidelines for Social Science Research in Health. <http://www.cehat.org/go/uploads/EthicalGuidelines/ethicalguidelines.pdf>. Accessed 15 June 2020.

Cleaton-Jones, P., & Wassenaar, D. (2010). Protection of human participants in health research - a comparison of some US Federal Regulations and South African Research Ethics guidelines. *South African Medical Journal*, 100(11), 712–716.

Council of Science Editors (CSE). (2018). CSE's White Paper on Promoting Integrity in Scientific Journal Publications. <https://druwt19tzv6d76es3lg0qdo7-wpengine.netdna-ssl.com/wp-content/uploads/CSE-White-Paper_2018-update-050618.pdf>. Accessed 15 June 2020.

Dade, A., Olafson, L., & Moody, N. (2015). Part II: Research Integrity: Conflict of Interest and Commitment in Research. In: A. Dade, O. Lori, & S. M. DiBella (Eds.), *Implementing a Comprehensive Research Compliance Program: A Handbook for Research Officers* (pp. 123–144). Charlotte, NC: Information Age Publishing, Inc.

Danish Committees on Scientific Dishonesty. (2009). Chapter 4: Guidelines relating to rights and duties concerning storage and use of research data. In: The Danish Committees on Scientific Dishonesty. *Guidelines for Good Scientific Practice* (pp. 23–30). [https://ufm.dk/en/publications/2009/files-2009/historical-guidelines-for-good-scientific-practice.pdf. Accessed 17 June 2020](https://ufm.dk/en/publications/2009/files-2009/historical-guidelines-for-good-scientific-practice.pdf.%20Accessed%2017%20June%202020).

Danish Ministry of Higher Education and Science. (2014). Danish Code of Conduct for Research Integrity. <https://ufm.dk/en/publications/2014/files-2014-1/the-danish-code-of-conduct-for-research-integrity.pdf>. Accessed 17 June 2020.

Department of Health and Human Services (HHS). (2005). 42 CFR–Part 93 Public Health Service Policies on Research Misconduct. <https://ori.hhs.gov/sites/default/files/42_cfr_parts_50_and_93_2005.pdf>. Accessed 17 June 2020.

Department of Health and Human Services (HHS), Office for Human Research Protections (OHRP) [Internet]. Mini-Tutorials. <https://www.hhs.gov/ohrp/education-and-outreach/online-education/mini-tutorials/index.html>.

Deutsche Forschungsgemeinschaft (DFG). (2013). Proposals for Safeguarding Good Scientific Practice (2nd ed.). Weinheim: Wiley - VCH.

Duggins Peloso, E., Velahos Koch, A., & Canovas, J. (2015). Part III: Regulatory/Legal Issues: Compliance Issues in International Research and International Research Collaborations. In: A. Dade, O. Lori, & S. M. DiBella (Eds.), *Implementing a Comprehensive Research Compliance Program: A Handbook for Research Officers* (pp. 211–248). Charlotte, NC: Information Age Publishing, Inc.

Dwivedi, G., & Tripathi, M. (2017). Stemming misconduct in higher education and research. *Annals of Library and Information Studies*, 64(4) 282-284. <http://nopr.niscair.res.in/handle/123456789/43419>. Accessed 15 June 2020.

Eckstein, L., Chalmers, D., Critchley, C., Jeanneret, R., McWhirter, R., Nielsen, J., Otlowski, M., & Nicol, D. (2018). Australia: Regulating Genomic Data Sharing to Promote Public Trust. *Human genetics*, 137(8), 583–591. <https://doi.org/10.1007/s00439-018-1914-z>.

Eckstein, S. (Ed.). (2003). *Manual for Research Ethics Committees: Centre of Medical Law and Ethics*, King's College London. Cambridge: Cambridge University Press.

Environmental Protection Agency (EPA). (2003). Policy and Procedures for Addressing Research Misconduct. <https://ori.hhs.gov/sites/default/files/epapolicy.pdf>. Accessed 15 June 2020.

Epstein, M., A., & Lascher, S. (2015). Part I: Research Subject Protection: Human Research Protection Programs. In: A. Dade, O. Lori, & S. M. DiBella (Eds.), *Implementing a Comprehensive Research Compliance Program: A Handbook for Research Officers* (pp. 9–40). Charlotte, NC: Information Age Publishing, Inc.

Euro Scientist (2017). The Brussels declaration on ethics & principles for science & society policy-making. <http://www.euroscientist.com/wp-content/uploads/2017/02/Brussels-Declaration.pdf>. Accessed 15 June 2020.

European Commission (EC). (2005). The European Charter for Researchers. 2005. <https://euraxess.ec.europa.eu/sites/default/files/am509774cee_en_e4.pdf>. Accessed 15 June 2020.

European Forum for Good Clinical Practice (EFGCP). (1997). Guidelines and Recommendations

for European Ethics Committees. <http://www.jirb.org.tw/DB/File/Download/efgcp-gidelinesandrecom.pdf>. Accessed 15 June 2020.

European Medicines Agency (EMA). (2002). Guidelines for Good Clinical Practice. <https://www.ema.europa.eu/en/documents/scientific-guideline/ich-e6-r1-guideline-good-clinical-practice_en.pdf>. Accessed 15 June 2020.

European Network of Research Ethics and Research Integrity (ENERI) project [Internet]. ENERI Classroom: Training and Capacity-Building Resource. <https://eneri.mobali.com/>. Accessed 15 June 2020.

European Network of Research Ethics and Research Integrity (ENERI) project, European Network of Research Integrity Offices (ENRIO), & Austrian Agency for Research Integrity (OeAWI) (2019). ENRIO Handbook: Recommendations for the Investigation of Research Misconduct. <http://www.enrio.eu/wp-content/uploads/2019/03/INV-Handbook_ENRIO_web_final.pdf>. Accessed 16 June 2020.

European Science Foundation (ESF). (2000). Good scientific practice in research and scholarship. <http://archives.esf.org/fileadmin/Public_documents/Publications/ESPB10.pdf>. Accessed 16 June 2020.

European Science Foundation (ESF). (2011). Fostering Research Integrity in Europe: A report by the ESF Member Organisation Forum on Research Integrity. <https://www.esf.org/fileadmin/user_upload/esf/ResearchIntegrity_Report2011.pdf>. Accessed 16 June 2020.

Evans, I. (2000). The Medical Research Council's Approach to Allegations of Scientific Misconduct. *Science and Engineering Ethics*, 6(1), 91–94. <https://doi.org/10.1007/s11948-000-0027-x>.

Fagot-Largeault, A. (2000). [Guidelines for clinical research: balance sheet on the law for biomedical research involving human subjects]. *Médecine/Sciences*, 16,1198–1202.

Fanelli, D. (2019a). European Commission. Research and Innovation Observatory-Horizon 2020 Policy Support Facility. MLE on Research Integrity: Thematic Report No 2 - Incentives. <https://rio.jrc.ec.europa.eu/policy-support-facility/mle-research-integrity>. Accessed 16 June 2020.

Fanelli, D. (2019b). European Commission. Research and Innovation Observatory-Horizon 2020 Policy Support Facility. MLE on Research Integrity: Thematic Report No 4 - Training and Education. <https://rio.jrc.ec.europa.eu/policy-support-facility/mle-research-integrity>. Accessed 16 June 2020.

Finnish National Board on Research Integrity (TENK). (2012). Finnish Advisory Board on Research Integrity: Responsible conduct of research and procedures for handling allegations of misconduct in Finland. <https://www.tenk.fi/sites/tenk.fi/files/HTK_ohje_2012.pdf>. Accessed 16 June 2020.

Foeger, N. & Zimmerman, S. (2016). Research Integrity: Perspectives from Austria and Canada. In T. Bretag (Ed.), *Handbook of Academic Integrity* (pp. 809–821). Singapore: Springer.

Forsberg, E., Anthun, F. O., Bailey, S., Birchley, G., Bout, H., Casonato, C. et al. (2018). Working with Research Integrity – Guidance for Research Performing Organisations: The Bonn PRINTEGER Statement. *Science and Engineering Ethics*, 24, 1023–1034. <https://doi.org/10.1007/s11948-018-0034-4>.

Global Research Council (GRC). (2013). Statement of Principles on Research Integrity. <https://www.globalresearchcouncil.org/grc-publications/>. Accessed 16 June 2020.

Graf, C., Battisti, W. P., Bridges, D., Bruce-Winkler, V., Conaty, J. M., Ellison, J. M. et al. (2009). Research Methods & Reporting. Good publication practice for communicating company sponsored medical research: the GPP2 guidelines. *BMJ (Clinical research ed.)*, 339, b4330. <https://doi.org/10.1136/bmj.b4330>.

Harvard Medical School. (2005). Principles and Procedures for Dealing with Allegations of Faculty Misconduct. <https://ari.hms.harvard.edu/sites/g/files/mcu761/files/principles_and_procedures_for_dealing_with_allegations_of_faculty_misconduct.pdf>. Accessed 16 June 2020.

Hausbeck Korgan, K. (2015). Part II: Research Integrity, Transparency, and Trust: Fostering Research Integrity and Compliance in Graduate Education. In: A. Dade, O. Lori, & S. M. DiBella (Eds.), *Implementing a Comprehensive Research Compliance Program: A Handbook for Research Officers* (pp. 181–208). Charlotte, NC: Information Age Publishing, Inc.

Hendrickson, T. L. (2015). Integrating responsible conduct of research education into undergraduate biochemistry and molecular biology laboratory curricula. *Biochemistry and molecular biology education: a bimonthly publication of the International Union of Biochemistry and Molecular Biology*, 43(2), 68–75. <https://doi.org/10.1002/bmb.20857>.

Hicks, D., Wouters, P., Waltman, L., de Rijcke, S., & Rafols, I. (2015). Bibliometrics: The Leiden Manifesto for research metrics. *Nature*, 520(7548), 429–431. <https://doi.org/10.1038/520429a>.

Hiney, M. (2015). Briefing Paper on Research Integrity: What it Means, Why it Is Important and How we Might Protect it. Science Europe: Science Europe Working Group on Research Integrity. <https://www.scienceeurope.org/our-resources/briefing-paper-on-research-integrity-what-it-means-why-it-is-important-and-how-we-might-protect-it>. Accessed 16 June 2020.

Institute of Medicine and National Research Council. (2002). *Integrity in Scientific Research: Creating an Environment That Promotes Responsible Conduct*. Washington, DC: The National Academies Press. <https://doi.org/10.17226/10430>.

Inter Academy Council (IAC), & Inter Academy Partners (IAP). (2012). Responsible Conduct in the Global Research Enterprise: A Policy Report. <https://www.interacademies.org/publication/responsible-conduct-global-research-enterprise>. Accessed 17 June 2020.

Irish Universities Association (IUA). (2014). National Policy Statement on Ensuring Research Integrity in Ireland. <https://www.iua.ie/publications/national-policy-statement-on-ensuring-research-integrity-in-ireland/>. Accessed 17 June 2020.

Israel, M., & Drenth, P. (2016). Research Integrity: Perspectives from Australia and Netherlands. In T. Bretag (Ed.), *Handbook of Academic Integrity* (pp. 789–808). Singapore: Springer.

Kyoto University. (2014). Kyoto University Regulations for Conflict of Interest Management Regulations. <https://www.kyoto-u.ac.jp/en/research/research-compliance-ethics/conflict_of_interest>. Accessed 16 June 2020.

Kyoto University. (2015). Promoting Research Integrity Regulations of Kyoto University. <https://www.kyoto-u.ac.jp/en/research/research-compliance-ethics/research-integrity-rules-reporting/documents/research-integrity-regulations201503.pdf>. Accessed 16 June 2020.

Kyoto University. (2019). Research Integrity leaflet ‘Responsible Academic Research’. <https://www.kyoto-u.ac.jp/en/research/research-compliance-ethics/research-integrity-rules-reporting/research-integrity-rules-reporting.html>. Accessed 16 June 2020.

Lerouge, I., & Hol, A. (2020). Towards a Research Integrity Culture at Universities: From Recommendations to Implementation. LERU publications. <https://www.leru.org/files/Towards-a-Research-Integrity-Culture-at-Universities-full-paper.pdf>. Accessed 16 June 2020.

Lo, B., & Field, M. J. (Eds.). (2009). *Conflict of Interest in Medical Research, Education, and Practice*. Washington, DC: The National Academies Press.

Marušić, A. (2019a). European Commission. Research and Innovation Observatory-Horizon 2020 Policy Support Facility. MLE on Research Integrity: Thematic Report No 3 - Dialogue and Communication. <https://rio.jrc.ec.europa.eu/policy-support-facility/mle-research-integrity>. Accessed 16 June 2020.

Marušić, A. (2019b). European Commission. Research and Innovation Observatory-Horizon 2020 Policy Support Facility. MLE on Research Integrity: Thematic Report No 1 - Processes and structures. <https://rio.jrc.ec.europa.eu/policy-support-facility/mle-research-integrity>. Accessed 16 June 2020.

McIntosh, N., Bates, P., Brykczynska, G., Dunstan, G., Goldman, A., Harvey, D. et al. (2000). Guidelines for the ethical conduct of medical research involving children. Royal College of Paediatrics, Child Health: Ethics Advisory Committee. *Archives of Disease in Childhood*, 82(2), 177–182. <https://doi.org/10.1136/adc.82.2.177>.

Medical University of Graz. (2014). Standards of Good Scientific Practice and Ombuds Committee at the Medical University of Graz.

<https://www.medunigraz.at/en/qualitaetsmanagement-in-der-forschung/good-scientific-practice/>. Accessed 16 June 2020.

Ministry of Education, Culture, Sports, Science and Technology, Japan (MEXT). (2014). Guidelines for Responding to Misconduct in Research. <https://www.mext.go.jp/a_menu/jinzai/fusei/1359618.htm>. Accessed 17 June 2020.

Moher, D., Bouter, L., Kleinert, S., Glasziou, P., Sham, M. H., Barbour, V. et al. (2019). The Hong Kong Principles for Assessing Researchers: Fostering Research Integrity. <https://doi.org/10.31219/osf.io/m9abx>. Accessed 17 June 2020.

Montreal Statement on Research Integrity in Cross-Boundary Research Collaborations. (2013). <https://wcrif.org/montreal-statement/file>. Accessed 17 June 2020.

Moodie, P. C., & Marshall, T. (1992). Guidelines for local research ethics committees. *BMJ (Clinical research ed.)*, 304(6837), 1293–1295. <https://doi.org/10.1136/bmj.304.6837.1293>.

Morris, S. E. (2010). Cracking the Code: Assessing Institutional Compliance with the Australian Code for the Responsible Conduct of Research. *Australian Universities’ Review*, 52(2), 18–26. <https://www.aur.org.au/archive/2010s>. Accessed 17 June 2020.

Nanyang Technological University (NTU). (2008). NTU Research Integrity Policy and Procedures. <https://www3.ntu.edu.sg/Research2/ResearchIntegrityPolicy.pdf>. Accessed 17 June 2020.

National Academies of Sciences, Engineering, and Medicine (NASEM). (2017). *Fostering Integrity in Research*. Washington, DC: The National Academies Press. <https://doi.org/10.17226/21896>. Accessed 17 June 2020.

National Academies of Sciences, Engineering, and Medicine (NASEM). (2018a). *Open Science by Design: Realizing a Vision for 21st Century Research*. Washington, DC: The National Academies Press. <https://doi.org/10.17226/25116>. Accessed 17 June 2020.

National Academies of Sciences, Engineering, and Medicine (NASEM). (2018b). *The Next Generation of Biomedical and Behavioral Sciences Researchers: Breaking Through*. Washington, DC: The National Academies Press. <https://doi.org/10.17226/25008>. Accessed 17 June 2020.

National Academies of Sciences, Engineering, and Medicine (NASEM). (2019). *Reproducibility and Replicability in Science*. Washington, DC: The National Academies Press. <https://doi.org/10.17226/25303>. Accessed 17 June 2020.

National Academy of Engineering (NAE). (2013). *Practical Guidance on Science and Engineering Ethics Education for Instructors and Administrators: Papers and Summary from a Workshop December 12, 2012.* Washington, DC: The National Academies Press. <https://doi.org/10.17226/18519>. Accessed 17 June 2020.

National Academy of Sciences, National Academy of Engineering, and Institute of Medicine. (1992). *Responsible Science: Ensuring the Integrity of the Research Process: Volume* *I*. Washington, DC: The National Academies Press. <https://doi.org/10.17226/1864>. Accessed 17 June 2020.

National Academy of Sciences, National Academy of Engineering, and Institute of Medicine. (1993). *Responsible Science: Ensuring the Integrity of the Research Process: Volume II*. Washington, DC: The National Academies Press. <https://doi.org/10.17226/2091>. Accessed 17 June 2020.

National Academy of Sciences, National Academy of Engineering, and Institute of Medicine. (2009a). *Ensuring the Integrity, Accessibility, and Stewardship of Research Data in the Digital Age*. Washington, DC: The National Academies Press. <https://doi.org/10.17226/12615>. Accessed 17 June 2020.

National Academy of Sciences, National Academy of Engineering, and Institute of Medicine. (2009b). *On Being a Scientist: A Guide to Responsible Conduct in Research: Third Edition*. Washington, DC: The National Academies Press. <https://doi.org/10.17226/12192>. Accessed 17 June 2020.

National Health and Medical Research Council (NHMRC), Australian Research Council, & Universities Australia. (2018a). *Australian Code for the Responsible Conduct of Research*. Canberra: National Health and Medical Research Council. <https://www.nhmrc.gov.au/about-us/publications/australian-code-responsible-conduct-research-2018>. Accessed 17 June 2020.

National Health and Medical Research Council (NHMRC), Australian Research Council, & Universities Australia. (2018b). *National Statement on Ethical Conduct in Human Research 2007 (updated 2018)*. Canberra: National health and Medical Research Council.

<https://www.nhmrc.gov.au/about-us/publications/national-statement-ethical-conduct-human-research-2007-updated-2018#block-views-block-file-attachments-content-block-1>. Accessed 17 June 2020.

National Health and Medical Research Council (NHMRC), Australian Research Council, & Universities Australia. (2019a). *Authorship: A guide supporting the Australian Code for the Responsible Conduct of Research*. Canberra: National Health and Medical Research Council. <https://www.nhmrc.gov.au/about-us/publications/australian-code-responsible-conduct-research-2018>. Accessed 17 June 2020.

National Health and Medical Research Council (NHMRC), Australian Research Council, & Universities Australia. (2019b). *Disclosure of interest and management of conflicts of interest: A guide supporting the Australian Code for the Responsible Conduct of Research*. Canberra: National Health and Medical Research Council.

<https://www.nhmrc.gov.au/about-us/publications/australian-code-responsible-conduct-research-2018>. Accessed 17 June 2020.

National Health and Medical Research Council (NHMRC), Australian Research Council, & Universities Australia. (2019c). *Management of Data and Information in Research: A guide supporting Australian Code for the Responsible Conduct of Research*. Canberra: National Health and Medical Research Council.

<https://www.nhmrc.gov.au/about-us/publications/australian-code-responsible-conduct-research-2018>. Accessed 17 June 2020.

National Health and Medical Research Council (NHMRC), Australian Research Council, & Universities Australia. (2019d). *Peer Review: A guide supporting the Australian Code for Responsible Conduct of Research*. Canberra: National Health and Medical Research Council. <https://www.nhmrc.gov.au/about-us/publications/australian-code-responsible-conduct-research-2018>. Accessed 17 June 2020.

National Health Research Ethics Committee (NHREC). (2007). *National Code of Health Research Ethics*. Abuja: Federal Ministry of Health. <http://www.nhrec.net/nhrec/NCHRE_Aug%2007.pdf>. Accessed 17 June 2020.

National Institutes of Health (NIH). (2011). Update on the Requirement for Instruction in the Responsible Conduct of Research. <https://grants.nih.gov/grants/guide/notice-files/not-od-10-019.html>. Accessed 17 June 2020.

National Science Foundation (NSF). (2002). Code of Federal Regulations Title 45, Subtitle B, Chapter VI, Part 689 – Research Misconduct. <https://www.nsf.gov/oig/_pdf/cfr/45-CFR-689.pdf>. Accessed 17 June 2020.

Nepal Health Research Council (NHRC). (2011). *National Ethical Guidelines For Health Research in Nepal And Standard Operating Procedures*. Ramshah Path: Nepal Health Research Council. <http://nhrc.gov.np/wp-content/uploads/2017/02/National_Ethical_Guidelines.pdf>. Accessed 17 June 2020.

Netherlands Code of Conduct for Research Integrity. (2018). <https://www.nwo.nl/en/policies/scientific+integrity+policy/netherlands+code+of+conduct+for+research+integrity>. Accessed 17 June 2020.

Nys, H. (2012). New European Rules Regarding the Approval of Clinical Trials, the Role of Ethics Committees and the Protection of Subjects. *Archivum immunologiae et therapiae experimentalis*, 60(6), 405–414. <https://doi.org/10.1007/s00005-012-0200-3>.

Office of Science and Technology Policy (OSTP). (2000). Federal Research Misconduct Policy. <https://ori.hhs.gov/federal-research-misconduct-policy>. Accessed 17 June 2020.

Organisation for Economic Co-operation and Development (OECD) Global Science Forum. (2007). Best Practices for Ensuring Scientific Integrity and Preventing Misconduct. <http://www.oecd.org/science/inno/40188303.pdf>. Accessed 17 June 2020.

Organisation for Economic Co-operation and Development (OECD) Global Science Forum. (2009). Investigating Research Misconduct Allegations in International Collaborative Research projects: A Practical Guide. <http://www.oecd.org/science/inno/42770261.pdf>. Accessed 24 June 2020.

Parder, M., & Juurik M. (2019). Promoting ethics and integrity in non-medical research (PRO-RES) project. Reporting on existing Codes and Guidelines. <http://prores-project.eu/wp-content/uploads/2019/12/D1_Existing_Code_and_guidelines.pdf>. Accessed 17 June 2020.

Penders, B., Shaw, D., Lutz, P., Townend, D., Akrong, L., & Zvonareva, O. (2018). ENERI Manual: Research Integrity and Ethics. <http://eneri.eu/reri-manual/>. Accessed 15 June 2020.

Pharmaceutical Research and Manufacturers of America (PhRMA). (2014). Principles on Conduct of Clinical Trials: Communication of Clinical Trials Results. Washington, DC: Pharmaceutical Research and Manufacturers of America. <https://www.phrma.org/en/Codes-and-guidelines/PhRMA-Principles-on-Conduct-of-Clinical-Trials>. Accessed 17 June 2020.

Piasecki, J., Waligora, M., & Dranseika, V. (2017). What Do Ethical Guidelines for Epidemiology Say About an Ethics Review? A Qualitative Systematic Review. *Science and Engineering Ethics*, 23(3), 743–768. <https://doi.org/10.1007/s11948-016-9829-3>.

Queen's University Belfast (QUB). (2014). QUB Code of Conduct and Integrity in Research. <https://www.qub.ac.uk/Research/Governance-ethics-and-integrity/Policies-procedures-and-guidelines/>. Accessed 17 June 2020.

Resnik, D. B., & Shamoo, A. E. (2011). The Singapore Statement on Research Integrity. *Accountability in research*, 18(2), 71–75. <https://doi.org/10.1080/08989621.2011.557296>.

Royal College of Physicians. (2007). *Guidelines on the practice of ethics committees in medical research with human participants (4th ed.)*. London: Royal College of Physicians.

Royal Netherlands Academy of Arts and Sciences (KNAW). (2008). *A Code of Conduct for Biosecurity: Report by the Biosecurity Working Group*. Amsterdam: Royal Netherlands Academy of Arts and Sciences.

Royal Netherlands Academy of Arts and Sciences (KNAW). (2013). *Responsible Research Data Management and the Prevention of Scientific Misconduct*. Amsterdam: Royal Netherlands Academy of Arts and Sciences.

Sallans, A. L., & Patterson, R. J. (2015). Part V: Related Compliance Issues: Data Management. In: A. Dade, O. Lori, & S. M. DiBella (Editors), *Implementing a Comprehensive Research Compliance Program: A Handbook for Research Officers* (pp. 477–494). Charlotte, NC: Information Age Publishing, Inc.

San Francisco Declaration on Research Assessment (DORA). (2012). <https://sfdora.org/read/>. Accessed 17 June 2020.

Schaller-Demers, D. (2015). Part II: Research Integrity: Building a Responsible Conduct of Research Program to Sustain an Institutional Culture of Research Integrity and Compliance. In: A. Dade, O. Lori, & S. M. DiBella (Eds.), *Implementing a Comprehensive Research Compliance Program: A Handbook for Research Officers* (pp. 71–100). Charlotte, NC: Information Age Publishing, Inc.

Science Council of Japan (SCJ). (2006). Code of Conduct for Scientists. <http://www.scj.go.jp/en/report/code.html>. Accessed 17 June 2020.

Science Europe. (2013). Humanities Scientific Committee Opinion Paper: Open Access Opportunities for the Humanities. <https://www.scienceeurope.org/our-resources/open-access-opportunities-for-the-humanities/>. Accessed 17 June 2020.

Science Europe. (2015). Science Europe Principles on Open Access to Research Publications. <https://www.scienceeurope.org/our-resources/principles-on-open-access-to-research-publications/>. Accessed 17 June 2020.

Science Europe. (2017). Workshop Report: Advancing Research Integrity Practices and Policies: From Recommendation to Implementation. <https://www.scienceeurope.org/our-resources/advancing-research-integrity-practices-and-policies-from-recommendation-to-implementation/>. Accessed 17 June 2020.

Science Europe. (2018a). Guidance document: Presenting a Framework for Discipline-specific

Research Data Management. <http://www.scienceeurope.org/our-resources/guidance-document-presenting-a-framework-for-discipline-specific-research-data-management>. Accessed 17 June 2020.

Science Europe. (2018b). Practical Guide to the International Alignment of Research Data Management. <https://www.scienceeurope.org/our-resources/practical-guide-to-the-international-alignment-of-research-data-management/>. Accessed 17 June 2020.

Science Europe. (2020). Implementing Research Data Management Policies across Europe: Experiences from Science Europe Member Organisations. <https://www.scienceeurope.org/our-resources/implementing-research-data-management-policies-across-europe/>. Accessed 17 June 2020.

Secretariat on Responsible Conduct of Research (2016). Tri-Agency Framework: Responsible Conduct of Research (cat. no. RR4-1/2016). <https://rcr.ethics.gc.ca/eng/documents/Framework2016-CadreReference2016_eng.pdf>. Accessed 15 June 2020.

Shimokai, H., Hata, S., Tamura, T., Yano, Y., Abe, S., & Takezawa, M. et al. (2007). The JSQA guideline for GCP auditing. *The Quality Assurance Journal*, 11(1), 37–43. <https://doi.org/10.1002/qaj.403>.

South African Medical Research Council (SAMRC). (2018). The South African Medical Research Council Guidelines on the Responsible Conduct of Research. <https://www.samrc.ac.za/research/ethics/guideline-documents>. Accessed 17 June 2020.

Spanish National Research Council (CSIC). (2015). National Statement on Scientific Integrity. <http://www.enrio.eu/wp-content/uploads/2017/03/csic-national-statement-on-scientific-integrity.pdf>. Accessed 17 June 2020.

Stakeholders Acting Together On the ethical impact assessment of Research and Innovation (SATORI) project. (2017). Policy Brief: Improving the organisation of research ethics committees (RECs). <https://satoriproject.eu/publications/improving-the-organisation-of-research-ethics-committees-recs/>. Accessed 17 June 2020.

The National Committee for Research Ethics in Science and Technology (NENT). (2016). *Guidelines for Research Ethics in Science and Technology (2nd ed.)*. Oslo: The Norwegian National Research Ethics Committees. <https://www.etikkom.no/en/ethical-guidelines-for-research/guidelines-for-research-ethics-in-science-and-technology/>. Accessed 17 June 2020.

The National Committee for Research Ethics in Social Sciences and the Humanities (NESH). (2016). *Guidelines for Research Ethics in the Social Sciences, Humanities, Law and Theology (4th ed.)*. Oslo: The Norwegian National Research Ethics Committees. <https://www.etikkom.no/en/ethical-guidelines-for-research/guidelines-for-research-ethics-in-the-social-sciences--humanities-law-and-theology/>. Accessed 17 June 2020.

The Office of Research Integrity (ORI). (1995). ORI Guidelines for Institutions and Whistleblowers: Responding to Possible Retaliation Against Whistleblowers in Extramural Research. <https://ori.hhs.gov/sites/default/files/2017-12/guidelines_whistle.pdf>. Accessed 17 June 2020.

The Office of Research Integrity (ORI). (1998). Tips for Sequestration of Physical Evidence in Research Misconduct Cases. <https://ori.hhs.gov/tips-for-sequestration>. Accessed 17 June 2020.

Toom, K., & Miller, P. F. (2018). Ethics and Integrity. In: J. Andersen, K. Toom, & S. Poli, *Research Management: Europe and Beyond* (pp. 264–286). London: Academic Press, Elsevier Inc.

Transparify [Internet]. <https://www.transparify.org/get-five>. Accessed 17 June 2020.

United Kingdom Health Ministers’ Gene Therapy Advisory Committee. (1995). Guidance on making proposals to conduct gene therapy research on human subjects. Report of the United Kingdom Health Ministers' Gene Therapy Advisory Committee. (1995). *Human Gene Therapy*, 6(3), 335–346. <https://doi.org/10.1089/hum.1995.6.3-335>.

United Kingdom Research Integrity Office (UKRIO). (2008). Procedure for the investigation of misconduct in research. <https://ukrio.org/wp-content/uploads/UKRIO-Procedure-for-the-Investigation-of-Misconduct-in-Research.pdf>. Accessed 17 June 2020.

United Kingdom Research Integrity Office (UKRIO). (2009). Code of Practice for Research: Promoting good practice and preventing misconduct. <https://ukrio.org/wp-content/uploads/UKRIO-Code-of-Practice-for-Research.pdf>. Accessed 17 June 2020.

Universities UK. (2019). The Concordat to Support Research Integrity. <https://www.universitiesuk.ac.uk/policy-and-analysis/reports/Documents/2019/the-concordat-to-support-research-integrity.pdf>. Accessed 18 June 2020.

University of Oxford. Policy on the Management of Data Supporting Research Outputs [Internet]. <https://researchdata.ox.ac.uk/university-of-oxford-policy-on-the-management-of-data-supporting-research-outputs/>. Accessed 18 June 2020.

University of Oxford. (2018a). Code of practice and procedure on academic integrity in research. <https://hr.admin.ox.ac.uk/academic-integrity-in-research#collapse1316006>. Accessed 18 June 2020.

University of Oxford. (2018b). Data protection policy. <https://compliance.admin.ox.ac.uk/data-protection-policy#collapse1172256>. Accessed 18 June 2020.

University of Oxford. (2019a). Conflict of interest policy. <https://researchsupport.admin.ox.ac.uk/governance/integrity/conflict/policy>. Accessed 18 June 2020.

University of Oxford. (2019b). Policy on the ethical research involving human participants and personal data. <https://researchsupport.admin.ox.ac.uk/governance/ethics/committees/policy#collapse395121>. Accessed 18 June 2020.

University of Southern Queensland (USQ). (2018). Animal Ethics Committee Procedure. <https://policy.usq.edu.au/documents/141878PL>. Accessed 18 June 2020.

University of Southern Queensland (USQ). (2019). Conflict of Interest Policy. <https://policy.usq.edu.au/documents/142758PL>. Accessed 18 June 2020.

University of Tartu, Centre for Ethics, & Estonian Research Council. (2017). Estonian Code of Conduct for Research Integrity. Tartu: Centre for Ethics, University of Tartu. <https://www.eetika.ee/sites/default/files/www_ut/hea_teadustava_eng_trukis.pdf>. Accessed 18 June 2020.

Van Andel, R. (2015). Part I: Research Subject Protection: Animal Care and Use. In: A. Dade, O. Lori, & S. M. DiBella (Eds.), *Implementing a Comprehensive Research Compliance Program: A Handbook for Research Officers* (pp. 41–67). Charlotte, NC: Information Age Publishing, Inc.

Wager, E., & Kleinert, S. on behalf of COPE Council. (2012). Cooperation between research institutions and journals on research integrity cases: guidance from the Committee on Publication Ethics (COPE). <https://publicationethics.org/files/Research_institutions_guidelines_final_0_0.pdf>. Accessed 18 June 2020.

Wellcome Trust. (2018). Good research practice guidelines. <https://wellcome.ac.uk/grant-funding/guidance/good-research-practice-guidelines>. Accessed 18 June 2020.

World Economic Forum (WEF). (2018). Code of Ethics. <http://www3.weforum.org/docs/WEF_Code_of_Ethics.pdf>. Accessed 18 June 2020.

World Health Organization (WHO). (1995). Guidelines for good clinical practice (GCP) for trials on pharmaceutical products.

<http://www.femh-irb.org/content_pages/files_add/doc_arb/I01_9712011000.pdf>. Accessed 18 June 2020.

World Health Organization (WHO). (2005). Handbook for Good Clinical Research Practice (GCP): guidance for implementation. <https://apps.who.int/iris/bitstream/handle/10665/43392/924159392X_eng.pdf?sequence=1&isAllowed=y>. Accessed 18 June 2020.

World Medical Association (WMA). (2018). WMA Declaration of Helsinki - Ethical Principles for Medical Research Involving Human Subjects. <https://www.wma.net/policies-post/wma-declaration-of-helsinki-ethical-principles-for-medical-research-involving-human-subjects/>. Accessed 18 June 2020.
