# Supplementary material for: Practices for Research Integrity Promotion in Research Performing Organisations and Research Funding Organisations: A Scoping Review
Source: Sci Eng Ethics. 2021 Jan 27;27(1):4. doi: 10.1007/s11948-021-00281-1 (PMC7840650; doi:10.1007/s11948-021-00281-1)
Supplement: Supplementary file 5 — Supplementary material 5 (DOCX 50 kb) [file 11948_2021_281_MOESM5_ESM.docx]

**Appendix 5 The list of practices aimed at individual researchers classified by research processes and RI topics**

| **Research process** | **RI topics** |
| --- | --- |
| **Research planning** | **Applying for financial resources** (University of Tartu 2017; SAMRC 2018; Path2Integrity)  **Authorship and publication plan** (National Academy of Sciences, National Academy of Engineering, and Institute of Medicine 1993; Albert and Wager 2003; Danish Committees on Scientific Dishonesty 2009a; Graf et al. 2009; IADR 2009; UKRIO 2009; ESF 2011; Swiss Academies of Arts and Sciences 2013; University of Wollongong 2017; Wellcome Trust 2018; Matheson 2019; NHMRC 2019a; USQ 2020)  **Consideration of ethical issues (including risk-benefit assessment)** (United Kingdom Health Ministers 1995; CEHAT 2000; Mcintosh et al. 2000; Eckstein 2003; RCN 2004; UNESCO 2004; EC 2005; Korenman 2006; Macrina 2007; Royal College of Physicians 2007; KNAW 2008; UKRIO 2009; NHRC 2011; Resnik and Shamoo 2011; Wager and Kleinert 2011; Medical University of Vienna 2013; WMA 2013; Danish Ministry of Higher Education and Science 2014; PhRMA 2014; NENT 2016; The National Committee for Research Ethics on Human Remains 2016; ALLEA 2017; University of Tartu 2017; Borgeat et al. 2018; Eckstein et al. 2018; NENT 2018; Netherlands Code of Conduct for Research Integrity 2018; Penders et al. 2018; TRUST 2018; Wellcome Trust 2018; Parder and Juurik 2019; Universities UK 2019; ENERI decision tree; Path2Integrity)  **Research methodology** (National Academy of Sciences, National Academy of Engineering, and Institute of Medicine 1993; Panacek and Lewis 1995; Doherty and Van de Putte 2000; HSRC 2006; Korenman 2006; Northern Illinois University 2006b; Royal College of Physicians 2007; UKRIO 2009; ESF 2011; Resnik and Shamoo 2011; Wager and Kleinert 2011; University of Utrecht 2014; CSIC 2015; Nebeker and Lopez-Arenas 2016; University of Tartu 2017; Netherlands Code of Conduct for Research Integrity 2018; Wellcome Trust 2018; Marušić 2019; NASEM 2019; Parder and Juurik 2019; ENERI decision tree)  **Research protocol (including DMP)** (Idänpään-Heikkilä 1994; ESF 2000; Cales et al. 2001; EMA 2002; NIH 2003; Bryn Mawr College 2004; Danish Committees on Scientific Dishonesty 2009a; NHRC 2011; Wager and Kleinert 2011; Medical University of Vienna 2013; WMA 2013; Danish Ministry of Higher Education and Science 2014; NWO 2016; ALLEA 2017; Borgeat et al. 2018; Netherlands Code of Conduct for Research Integrity 2018; Science Europe 2018a; DCC; Path2Integrity)  **Research registration** (WMA 2013; PhRMA 2014; Wellcome Trust 2018) |
| **Research conducting** | **Authorship** (Harvard Medical School 1991; Friedman 1993; National Academy of Sciences, National Academy of Engineering, and Institute of Medicine 1993; Holaday and Yost 1995; Harvard Medical School 1999; CEHAT 2000; Doherty and Van de Putte 2000; ESF 2000; Cales et al. 2001; Albert and Wager 2003; EC 2005; Macrina 2007; Graf et al. 2009; National Academy of Sciences, National Academy of Engineering, and Institute of Medicine 2009b; UKRIO 2009; Morris 2010; Resnik and Shamoo 2011; Wager and Kleinert 2011; Nichols-Casebolt 2012; DFG 2013; Medical University of Vienna 2013; Swiss Academies of Arts and Sciences 2013; Medical University of Graz 2014; PhRMA 2014; QUB 2014; ACS 2015; CSIC 2015; Hendrickson 2015; OeAWI 2015; EECERA 2015; Israel and Drenth 2016; Matheson 2016; NENT 2016; NESH 2016; ALLEA 2017; Santos et al. 2017; University of Tartu 2017; University of Wollongong 2017; CSE 2018; Netherlands Code of Conduct for Research Integrity 2018; Penders et al. 2018; SAMRC 2018; Matheson 2019; NHMRC 2019a; Parder and Juurik 2019; USQ 2020; ENERI decision tree; Nature; Path2Integrity)  **Collaboration** (IADR 2009; UKRIO 2009; Montreal Statement 2013; Hendrickson 2015; ALLEA 2017; NASEM 2017; University of Tartu 2017; Netherlands Code of Conduct for Research Integrity 2018; TRUST 2018; NASEM 2019; Parder and Juurik 2019; ENERI decision tree; Path2Integrity)  **Conflict of interest** (CIOMS 1991; National Academy of Sciences, National Academy of Engineering, and Institute of Medicine 1993; Gibinski 1998; CEHAT 2000; Doherty and Van de Putte 2000; Cales et al. 2001; Komesaroff 2005; HSRC 2006; Korenman 2006; SCJ 2006; Danish Committees on Scientific Dishonesty 2009a; Graf et al. 2009; IADR 2009; Lo and Field 2009; National Academy of Sciences, National Academy of Engineering, and Institute of Medicine 2009b; UKRIO 2009; ESF 2011; NHRC 2011; Resnik and Shamoo 2011; Wager and Kleinert 2011; Nichols-Casebolt 2012; Medical University of Vienna 2013; Saver 2013; Danish Ministry of Higher Education and Science 2014; PhRMA 2014; QUB 2014; CSIC Manual 2015; EECERA 2015; Hendrickson 2015; OeAWI 2015; Israel and Drenth 2016; NENT 2016; NESH 2016; ALLEA 2017; Santos et al. 2017; University of Tartu 2017; CSE 2018; Penders et al. 2018; SAMRC 2018; Toom and Miller 2018; NHMRC 2019b; Parder and Juurik 2019; Universities UK 2019; University of Oxford 2019a; USQ 2019; ENERI; ENERI decision tree; Nature)  **Data management** (Harvard Medical School 1991; National Academy of Sciences, National Academy of Engineering, and Institute of Medicine 1993; Idänpään-Heikkilä 1994; Panacek and Lewis 1995; Gibinski 1998; ESF 2000; Cales et al. 2001; EMA 2002; NIH 2003; Couleham and Wells 2006; SCJ 2006; Danish Committees on Scientific Dishonesty 2009b; National Academy of Sciences, National Academy of Engineering, and Institute of Medicine 2009a; National Academy of Sciences, National Academy of Engineering, and Institute of Medicine 2009b; UKRIO 2009; ESF 2011; Resnik and Shamoo 2011; University of Connecticut 2011; Nichols-Casebolt 2012; DFG 2013; KNAW 2013; Medical University of Vienna 2013; University of Oxford; IUA 2014; Medical University of Graz 2014; QUB 2014; Kreissl Lonfat et al. 2015; Kyoto University 2015; Nebeker and Lopez-Arenas 2016; OeAWI 2015; Israel and Drenth 2016; NESH 2016; ALLEA 2017; Aoki et al. 2017; Garcia Arenillas et al. 2017; University of Tartu 2017; Netherlands Code of Conduct for Research Integrity 2018; SAMRC 2018; Science Europe 2018a; Science Europe 2018b; Toom and Miller 2018; Wellcome Trust 2018; Marušić 2019; NHMRC 2019c; Parder and Juurik 2019; ENERI decision tree; Path2Integrity)  **Data protection (privacy and confidentiality)** (CIOMS 1991; Harvey 1994; United Kingdom Health Ministers 1995; Phillips 1999; CEHAT 2000; EMA 2002; Eckstein 2003; NIH 2003; Bryn Mawr College 2004; RCN 2004; HSRC 2006; NITO 2006; Royal College of Physicians 2007; University of Waikato 2008; UKRIO 2009; ESF 2011; NHRC 2011; Wager and Kleinert 2011; WMA 2013; Danish Ministry of Higher Education and Science 2014; IUA 2014; Medical University of Graz 2014; EECERA 2015; NENT 2016; NESH 2016; ALLEA 2017; Santos et al. 2017; University of Tartu 2017; Araki et al. 2018; Eckstein et al. 2018; Penders et al. 2018; SAMRC 2018; TRUST 2018; WEF 2018; Parder and Juurik 2019; University of Oxford 2018b; University of Oxford 2019b; ENERI; ENERI decision tree)  **Informed consent** (Harvey 1994; Idänpään-Heikkilä 1994; Nuffield Council on Bioethics 1995; United Kingdom Health Ministers 1995; Phillips 1999; CEHAT 2000; ESF 2000; Fagot-Largeault 2000; Mcintosh et al. 2000; EMA 2002; Eckstein 2003; Maschke 2003; Bryn Mawr College 2004; RCN 2004; UNESCO 2004; de Castilho and Kalil 2005; HSRC 2006; Korenman 2006; NITO 2006; NHREC 2007; Royal College of Physicians 2007; University of Waikato 2008; UKRIO 2009; NHRC 2011; University of Connecticut 2011; Wager and Kleinert 2011; Nys 2012; WMA 2013; PhRMA 2014; EECERA 2015; NESH 2016; Garcia Arenillas et al. 2017; Levy et al. 2017; Borgeat et al. 2018; Penders et al. 2018; Toom and Miller 2018; TRUST 2018; Parder and Juurik 2019; University of Oxford 2019b; ENERI; ENERI decision tree; I-CONSENT; Path2Integrity)  **Intellectual property** (National Academy of Sciences, National Academy of Engineering, and Institute of Medicine 1993; ESF 2000; EC 2005; Danish Committees on Scientific Dishonesty 2009a; National Academy of Sciences, National Academy of Engineering, and Institute of Medicine 2009b; UKRIO 2009; University of Connecticut 2011; Danish Ministry of Higher Education and Science 2014; QUB 2014; CSIC 2015; Israel and Drenth 2016; University of Tartu 2017; Crisan and Iacob 2018; Penders et al. 2018; Toom and Miller 2018; Wellcome Trust 2018; Parder and Juurik 2019; Path2Integrity)  **Management of resources** (UKRIO 2009; University of Connecticut 2011; QUB 2014; CSIC 2015; EECERA 2015; ALLEA 2017; Kyoto University 2018; Parder and Juurik 2019; Path2Integrity)  **Mentorship/supervision** (National Academy of Sciences, National Academy of Engineering, and Institute of Medicine 1992; National Academy of Sciences, National Academy of Engineering, and Institute of Medicine 1993; CEHAT 2000; EC 2005; UKRIO 2009; Nichols-Casebolt 2012; DFG 2013; Danish Ministry of Higher Education and Science 2014; IUA 2014; Medical University of Graz 2014; QUB 2014; CSIC 2015; Hendrickson 2015; Kyoto University 2015; OeAWI 2015; NESH 2016; ALLEA 2017; University of Tartu 2017; NASEM 2018b; Netherlands Code of Conduct for Research Integrity 2018; SAMRC 2018; WEF 2018; Wellcome Trust 2018; Parder and Juurik 2019; ENERI; Path2Integrity)  **Protection of research subjects** (CIOMS 1991; Idänpään-Heikkilä 1994; Nuffield Council on Bioethics 1995; CEHAT 2000; EMA 2002; Eckstein 2003; Maschke 2003; RCN 2004; UNESCO 2004; de Castilho and Kalil 2005; HSRC 2006; NITO 2006; SCJ 2006; NHREC 2007; Royal College of Physicians 2007; University of Waikato 2008; IADR 2009; UKRIO 2009; Cleaton-Jones and Wassenaar 2010; University of Connecticut 2011; Nys 2012; Medical University of Vienna 2013; WMA 2013; Medical University of Graz 2014; PhRMA 2014; EECERA 2015; NENT 2016; NESH 2016; ALLEA 2017; Garcia Arenillas et al. 2017; Santos et al. 2017; University of Tartu 2017; Crisan and Iacob 2018; Eckstein et al. 2018; NENT 2018; Netherlands Code of Conduct for Research Integrity 2018; Penders et al. 2018; SAMRC 2018; TRUST 2018; WEF 2018; Wellcome Trust 2018; Parder and Juurik 2019; ENERI; I-CONSENT; Nature; Path2Integrity) |
| **Research dissemination** | **Dialogue with public/society** (EC 2005; SCJ 2006; NENT 2016; NESH 2016; Euro Scientist 2017; Netherlands Code of Conduct for Research Integrity 2018; WEF 2018)  **Open science** (Panacek and Lewis 1995; ESF 2011; Resnik and Shamoo 2011; ICSU 2014; IUA 2014; QUB 2014; CSIC 2015; NENT 2016; NESH 2016; ALLEA 2017; NASEM 2017; Franck 2018; NASEM 2018a; Toom and Miller 2018; WEF 2018; Wellcome Trust 2018; NASEM 2019; Parder and Juurik 2019)  **Publication ethics** (CIOMS 1991; National Academy of Sciences, National Academy of Engineering, and Institute of Medicine 1993; CEHAT 2000; Albert and Wager 2003; UNESCO 2004; EC 2005; HSRC 2006; SCJ 2006; Royal College of Physicians 2007; University of Alabama 2008; National Academy of Sciences, National Academy of Engineering, and Institute of Medicine 2009b; ESF 2011; Wager and Kleinert 2011; DORA 2012; Swiss Academies of Arts and Sciences 2013; WMA 2013; Danish Ministry of Higher Education and Science 2014; ICSU 2014; QUB 2014; ACS 2015; CSIC 2015; Hiney 2015; OeAWI 2015; EECERA 2015; Matheson 2016; NENT 2016; NESH 2016; ALLEA 2017; Santos et al. 2017; University of Tartu 2017; Crisan and Iacob 2018; SAMRC 2018; NHMRC 2019c; Parder and Juurik 2019; Nature; Path2Integrity)  **Research reporting (manuscript, reporting guidelines)** (Equator Network; Harvard Medical School 1991; National Academy of Sciences, National Academy of Engineering, and Institute of Medicine 1993; Albert and Wager 2003; UNESCO 2004; Northern Illinois University 2006b; Christiansen et al. 2007; Graf et al. 2009; Wager and Kleinert 2011; Swiss Academies of Arts and Sciences 2013; Medical University of Graz 2014; University of Utrecht 2014; ACS 2015; Bossuyt et al. 2015; Matheson 2016; NESH 2016; NASEM 2019; Nature) |
| **Research evaluation** | **Ethics approval** (CIOMS 1991; Doherty and Van de Putte 2000; Fagot-Largeault 2000; Mcintosh et al. 2000; EMA 2002; Eckstein 2003; Maschke 2003; RCN 2004; Korenman 2006; NITO 2006; University of Waikato 2008; National Academy of Sciences, National Academy of Engineering, and Institute of Medicine 2009b; UKRIO 2009; Cleaton-Jones and Wassenaar 2010; NHRC 2011; Wager and Kleinert 2011; Nys 2012; Medical University of Vienna 2013; WMA 2013; Danish Ministry of Higher Education and Science 2014; Medical University of Graz 2014; PhRMA 2014; QUB 2014; NENT 2016; Levy et al. 2017; Penders et al. 2018; NENT 2018; Netherlands Code of Conduct for Research Integrity 2018; SAMRC 2018; Toom and Miller 2018; USQ 2018; University of Oxford 2019b; ENERI; HHS; Path2Integrity)  **Peer review** (National Academy of Sciences, National Academy of Engineering, and Institute of Medicine 1993; CEHAT 2000; Doherty and Van de Putte 2000; Northern Illinois University 2006a; UKRIO 2009; Resnik and Shamoo 2011; Wager and Kleinert 2011; Nichols-Casebolt 2012; DFG 2013; Medical University of Graz 2014; QUB 2014; Rockwell 2014; ACS 2015; CSIC 2015; Hendrickson 2015; Israel and Drenth 2016; NENT 2016; ALLEA 2017; University of Tartu 2017; CSE 2018; Netherlands Code of Conduct for Research Integrity 2018; Penders et al. 2018; SAMRC 2018; NASEM 2019; NHMRC 2019d; Parder and Juurik 2019; ENERI; Nature; Path2Integrity) |
| **RI violations and resolutions** | **Detrimental/questionable/poor research practices** (National Academy of Sciences, National Academy of Engineering, and Institute of Medicine 1992; National Academy of Sciences, National Academy of Engineering, and Institute of Medicine 2009b; KNAW 2013; IUA 2014; Medical University of Graz 2014; Hiney 2015; OeAWI 2015; ALLEA 2017; NASEM 2017; Netherlands Code of Conduct for Research Integrity 2018; Penders et al. 2018; Universities UK 2019; ENERI)  **Fabrication, falsification and plagiarism** (National Academy of Sciences, National Academy of Engineering, and Institute of Medicine 1992; CEHAT 2000; Cockcroft 2000; Doherty and Van de Putte 2000; EC 2005; Korenman 2006; IADR 2009; National Academy of Sciences, National Academy of Engineering, and Institute of Medicine 2009b; UKRIO 2009; ESF 2011; Wager and Kleinert 2011; University of Connecticut 2011; KNAW 2013; Medical University of Vienna 2013; IUA 2014; Medical University of Graz 2014; ACS 2015; EECERA 2015; Hiney 2015; Kyoto University 2015; OeAWI 2015; Roig 2015; NESH 2016; ALLEA 2017; Dwivedi and Tripathi 2017; NASEM 2017; Santos et al. 2017; Netherlands Code of Conduct for Research Integrity 2018; Penders et al. 2018; Toom and Miller 2018; University of Oxford 2018a; Universities UK 2019; ENERI; Nature)  **Reporting misconduct** (National Academy of Sciences, National Academy of Engineering, and Institute of Medicine 1992; National Academy of Sciences, National Academy of Engineering, and Institute of Medicine 1993; KNAW 2008; NTU 2008; IADR 2009; UKRIO 2009; Resnik and Shamoo 2011; University of Connecticut 2011; Danish Ministry of Higher Education and Science 2014; ICSU 2014; Kyoto University 2015; University of Tartu 2017; Netherlands Code of Conduct for Research Integrity 2018; University of Oxford 2018a; Wellcome Trust 2018; Marušić 2019; Parder and Juurik 2019; Universities UK 2019)  **Whistle-blowing protection** (National Academy of Sciences, National Academy of Engineering, and Institute of Medicine 1992; NTU 2008; DFG 2013; NENT 2016; Parder and Juurik 2019) |
| **RI promotion** | **Training and education** (National Academy of Sciences, National Academy of Engineering, and Institute of Medicine 1992; CEHAT 2000; Evans 2000; Alexander and Williams 2004; Macrina 2007; IADR 2009; National Academy of Sciences, National Academy of Engineering, and Institute of Medicine 2009b; UKRIO 2009; NIH 2011; Danish Ministry of Higher Education and Science 2014; Föger and Zimmerman 2016; ALLEA 2017; NASEM 2017; NASEM 2018b; NHMRC 2019c; Path2Integrity)  **Research culture** (National Academy of Sciences, National Academy of Engineering, and Institute of Medicine 1992; EC 2005; SCJ 2006; National Academy of Sciences, National Academy of Engineering, and Institute of Medicine 2009b; UKRIO 2009; Danish Ministry of Higher Education and Science 2014; Medical University of Graz 2014; QUB 2014; Wellcome Trust 2018; Parder and Juurik 2019; Path2Integrity) |

ACS – American Chemical Society; ALLEA – All European Academies; CEHAT – Centre for Enquiry into Health and Allied Themes; CIOMS – Council of International Organizations of Medical Sciences; CSE – Council of Science Editors; CSIC – Spanish National research Council; DCC – Digital Curation Centre; DFG – Deutsche Forschungsgemeinschaft; DORA – San Francisco Declaration on Research Assessment; EC – European Commission; EMA – European Medicines Agency; ENERI – European Network of Research Ethics and Research Integrity; ESF – European Science Foundation; HHS – Department of Health and Human Services (United States); HSRC – Human Sciences Research Council; IADR – International Association for Dental Research; ICSU – International Council for Science; IUA – Irish Universities Association; KNAW – Royal Netherlands Academy of Arts and Sciences; NASEM – National Academies of Sciences, Engineering and Medicine; NENT – National Committee for Research Ethics in Science and Technology (Norway); NESH – National Committee for Research Ethics in Social Sciences and Humanities (Norway); NIH – National Institutes of Health (United States); NITO – Norwegian Institute of Biomedical Science; NHMRC – National Health and Medical research Council (Australia); NHREC – National Health Research Ethics Committee (Nigeria); NHRC – Nepal Health Research Council; NWO – Dutch Research Council; OeAWI – Austrian Agency for Research Integrity; PhRMA – Pharmaceutical Research and Manufacturers of America; QUB – Queen's University Belfast; RCN – Royal College of Nurses; SAMRC – South African Medical Research Council; SCJ – Science Council of Japan; UK – United Kingdom; UKRIO – United Kingdom Research Integrity Office; UNESCO – United Nations Educational, Scientific and Cultural Organization; USQ – University of Southern Queensland; WEF – World Economic Forum; WMA – World Medical Association

**References**

Albert, T., & Wager, E. on behalf of COPE Council. (2003). How to handle authorship disputes: a guide for new researchers. Version 1. <https://doi.org/10.24318/cope.2018.1.1>. Accessed 18 June 2020.

Alexander, M., & Williams, W.R. (2004). A Guidebook for Teaching Selected Responsible Conduct of Research Topics to a Culturally Diverse Trainee Group. Rockville, MD: Office of Research Integrity. <https://ori.hhs.gov/images/ddblock/Alexander.RCR%20Guidebook.BW_.pdf>. Accessed 18 June 2020.

All European Academies (ALLEA). (2017). European Code of Conduct for Research Integrity. <https://allea.org/code-of-conduct/>. Accessed 17 June 2020.

American Chemical Society (ACS). (2015). Ethical Guidelines to Publication of Chemical Research. <https://pubs.acs.org/userimages/ContentEditor/1218054468605/ethics.pdf>. Accessed 18 June 2020. Aoki, T., Kajita, S., Akasaka, H., & Takeda, H. (July 9-13, 2017). Development and Deployment of Research Data Preservation Policy at a Japanese Research University in 2016. 6th IIAI International Congress on Advanced Applied Informatics (IIAI-AAI), Hamamatsu, Japan.

Araki, K., Masuzawa, Y., Takahashi, Y., & Nakayama, T. (2018). [The Japanese legal system and the applicability of laws and regulations on private information protection and research ethics relating to medical research]. *Japanese Journal of Public Health*, 65(12), 730–743. <https://europepmc.org/article/med/30587680>. Accessed 15 June 2020.

Austrian Agency for Research Integrity (OeAWI). (2015). Guidelines for good scientific practice. <https://oeawi.at/wp-content/uploads/2018/09/OeAWI_Brosch%C3%BCre_Web_2019.pdf>. Accessed 15 June 2020.

Borgeat Meza, M., Luengo-Charath, X., Arancibia, M., & Madrid, E. (2018). Council for International Organizations of Medical Sciences (CIOMS) Ethical Guidelines: advancements and unsolved topics in 2016 upgrade. *Medwave*, 18(2), e7208. <https://doi.org/10.5867/medwave.2018.02.7208>.

Bossuyt, P. M., Reitsma, J. B., Bruns, D. E., Gatsonis, C. A., Glasziou, P. P., Irwig, L., Lijmer, J. G., Moher, D., Rennie, D., de Vet, H. C., Kressel, H. Y., Rifai, N., Golub, R. M., Altman, D. G., Hooft, L., Korevaar, D. A., Cohen, J. F., & STARD Group (2015). STARD 2015: an updated list of essential items for reporting diagnostic accuracy studies. *BMJ (Clinical research ed.)*, 351, h5527. <https://doi.org/10.1136/bmj.h5527>.

Bryn Mawr College and the Massachusetts College of Pharmacy and Health Sciences. (2004). Ethics and Research in the Community. <https://ori.hhs.gov/bryn-mawr-college-and-massachusetts-college-pharmacy-and-health-sciences>. Accessed 19 June 2020.

Cales, P., Barbare, J., Marteau, P., Nouel, O., Sautereau , D., & Valla, D. (2001). Charte de déontologie en recherche Clinique: Étape II. *Gastroentérologie Clinique et Biologique*, 25(11). <https://www.em-consulte.com/en/article/98623>. Accessed 18 June 2020.

Centre for Enquiry into Health and Allied Themes (CEHAT). (2000). National Committee for Ethics in Social Science Research in Health (NCESSRH): Ethical Guidelines for Social Science Research in Health. <http://www.cehat.org/go/uploads/EthicalGuidelines/ethicalguidelines.pdf>. Accessed 15 June 2020.

Christiansen, S. L., Iverson, C., Flanagin, A., Livingston, E. H., Fischer, L., Manno, C. *et al*. (2007). *AMA Manual of Style: A Guide for Authors and Editors* (10th ed.). New York, NY: Oxford University Press.

Cleaton-Jones, P., & Wassenaar, D. (2010). Protection of human participants in health research - a comparison of some US Federal Regulations and South African Research Ethics guidelines*. South African Medical Journal*, 100(11), 712–716.

Cockcroft A. (2000). COPE guidelines on good publication practice. Committee on Publication Ethics. *Occupational and environmental medicine*, 57(8), 505. <https://doi.org/10.1136/oem.57.8.505>.

Couleham, M. B., & Wells, J. F. (2006). *Guidelines for Responsible Data Management in Scientific Research*. Rockville, MD: Office of Research Integrity. <https://ori.hhs.gov/images/ddblock/data.pdf>. Accessed 18 June 2020.

Council for International Organizations of Medical Sciences (CIOMS). (1991). International guidelines for ethical review of epidemiological studies. <https://cioms.ch/wp-content/uploads/2017/01/1991_INTERNATIONAL_GUIDELINES.pdf>. Accessed 19 June 2020.

Council of Science Editors (CSE). (2018). CSE's White Paper on Promoting Integrity in Scientific Journal Publications. <https://druwt19tzv6d76es3lg0qdo7-wpengine.netdna-ssl.com/wp-content/uploads/CSE-White-Paper_2018-update-050618.pdf>. Accessed 15 June 2020.

Crisan, O. & Iacob, S. (2018). Romanian Code of Pharmaceutical Deontology - A New Conception. *Farmacia*, 66(1), 187–196.

Danish Committees on Scientific Dishonesty. (2009a). Chapter 3: Guidelines for agreements at the initiation of research projects. In: *The Danish Committees on Scientific Dishonesty.* *Guidelines for Good Scientific Practice* (pp. 13–22). <https://ufm.dk/en/publications/2009/files-2009/historical-guidelines-for-good-scientific-practice.pdf>. Accessed 19 June 2020.

Danish Committees on Scientific Dishonesty. (2009b). Chapter 4: Guidelines relating to rights and duties concerning storage and use of research data. In: *The Danish Committees on Scientific Dishonesty. Guidelines for Good Scientific Practice* (pp. 23–30). <https://ufm.dk/en/publications/2009/files-2009/historical-guidelines-for-good-scientific-practice.pdf>. Accessed 19 June 2020.

Danish Ministry of Higher Education and Science. (2014). Danish Code of Conduct for Research Integrity. <https://ufm.dk/en/publications/2014/files-2014-1/the-danish-code-of-conduct-for-research-integrity.pdf>. Accessed 18 June 2020.

De Castilho, E. A., & Kalil, J. (2005). [Ethics and medical research: principles, guidelines, and regulations]. *Revista da Sociedade Brasileira de Medicina Tropical*, 38(4), 344–347. <https://doi.org/10.1590/s0037-86822005000400013>.

Department of Health and Human Services (HHS), Office for Human Research Protections (OHRP) [Internet].Mini-Tutorials. <https://www.hhs.gov/ohrp/education-and-outreach/online-education/mini-tutorials/index.html>.

Deutsche Forschungsgemeinschaft (DFG). (2013). Proposals for Safeguarding Good Scientific Practice (2nd ed.). Weinheim: Wiley - VCH.

<https://www.imprs-tp.mpg.de/80564/DFG_Recommendations_2013.pdf>. Accessed 18 June 2020.

Digital Curation Centre (DCC) [Internet]. DMPonline. <https://dmponline.dcc.ac.uk/>. Accessed 18 June 2020.

Doherty, M., & Van De Putte, L. B. (2000). Committee on Publication Ethics (COPE) guidelines on good publication practice. *Annals of the Rheumatic Diseases*, 59(6), 403–404. <https://doi.org/10.1136/ard.59.6.403>.

Dutch Research Council (NWO). (2016). Data Management Policy. <https://www.nwo.nl/en/policies/open+science/data+management>. Accessed 19 June 2020.

Dwivedi, G., & Tripathi, M. (2017). Stemming misconduct in higher education and research. Annals of Library and Information Studies, 64(4) 282-284. <http://nopr.niscair.res.in/handle/123456789/43419>. Accessed 15 June 2020.

Eckstein, L., Chalmers, D., Critchley, C., Jeanneret, R., McWhirter, R., Nielsen, J., Otlowski, M., & Nicol, D. (2018). Australia: Regulating Genomic Data Sharing to Promote Public Trust. *Human genetics*, 137(8), 583–591. <https://doi.org/10.1007/s00439-018-1914-z>.

Eckstein, S. (Ed.). (2003). *Manual for Research Ethics Committees: Centre of Medical Law and Ethics*, King's College London. Cambridge: Cambridge University Press.

Enhancing the Quality and Transparency of Health Research (Equator Network) [Internet]. <https://www.equator-network.org/>. Accessed 18 June 2020.

Ethics of informed consent in novel treatment including a gender perspective (I-CONSENT) project [Internet]. <https://i-consentproject.eu/results/>. Accessed 19 June 2020.

Euro Scientist (2017). The Brussels declaration on ethics & principles for science & society policy-making. <http://www.euroscientist.com/wp-content/uploads/2017/02/Brussels-Declaration.pdf>. Accessed 15 June 2020.

European Commission (EC). (2005). The European Charter for Researchers. <https://euraxess.ec.europa.eu/sites/default/files/am509774cee_en_e4.pdf>. Accessed 15 June 2020.

European Early Childhood Education Research Association (EECERA). (2015). EECERA Ethical Code for Early Childhood Researchers. <https://www.eecera.org/wp-content/uploads/2016/07/EECERA-Ethical-Code.pdf>. Accessed 15 June 2020.

European Medicines Agency (EMA). (2002). Guidelines for Good Clinical Practice. <https://www.ema.europa.eu/en/documents/scientific-guideline/ich-e6-r1-guideline-good-clinical-practice_en.pdf>. Accessed 15 June 2020.

European Network of Research Ethics and Research Integrity (ENERI) project [Internet]. ENERI Classroom: Training and Capacity-Building Resource. <https://eneri.mobali.com/>. Accessed 15 June 2020.

European Network of Research Ethics and Research Integrity (ENERI) project [Interent]. ENERI decision tree. <http://eneri.eu/decision-tree/>. Accessed 18 June 2020.

European Science Foundation (ESF). (2000). Good scientific practice in research and scholarship. <http://archives.esf.org/fileadmin/Public_documents/Publications/ESPB10.pdf>. Accessed 16 June 2020.

European Science Foundation (ESF). (2011). Fostering Research Integrity in Europe: A report by the ESF Member Organisation Forum on Research Integrity. <https://www.esf.org/fileadmin/user_upload/esf/ResearchIntegrity_Report2011.pdf>. Accessed 16 June 2020.

Evans, I. (2000). The Medical Research Council's Approach to Allegations of Scientific Misconduct. *Science and Engineering Ethics*, 6(1), 91–94. <https://doi.org/10.1007/s11948-000-0027-x>.

Fagot-Largeault, A. (2000). [Guidelines for clinical research: balance sheet on the law for biomedical research involving human subjects]. *Médecine/Sciences*, 16,1198–1202.

Foeger, N. & Zimmerman, S. (2016). Research Integrity: Perspectives from Austria and Canada. In T. Bretag (Ed.), *Handbook of Academic Integrity* (pp. 809–821). Singapore: Springer.

Franck, G. (2018). Train-the-trainer card game for Open Science training. <https://www.fosteropenscience.eu/node/2570>. Accessed 18 June 2020.

Friedman, P. J. (1993). Standards for authorship and publication in academic radiology. AUR Ad Hoc Committee on standards for the responsible conduct of research. *Investigative Radiology*, 28(10), 879–881. <https://doi.org/10.1097/00004424-199310000-0000>.

García Arenillas, M., Haj-Ali Saflo, O., & Sáenz de Tejada, M. (2017). [New Royal Decree on clinical trials: main implications for emergency medicine physicians who do research]. *Emergencias : revista de la Sociedad Espanola de Medicina de Emergencias*, 29(3), 194–201.

Gibiński K. (1998). [Good manners in science. A collection of rules and principles. The committee on ethics in science from the executive board of the Polish Academy of Science]. *Polskie Archiwum Medycyny Wewnetrznej*, 100(4), 388–402.

Graf, C., Battisti, W. P., Bridges, D., Bruce-Winkler, V., Conaty, J. M., Ellison, J. M. et al. (2009). Research Methods & Reporting. Good publication practice for communicating company sponsored medical research: the GPP2 guidelines. *BMJ (Clinical research ed.)*, 339, b4330. <https://doi.org/10.1136/bmj.b4330>.

Harvard Medical School. (1991). Guidelines for Investigators in Clinical Research. <https://ari.hms.harvard.edu/sites/g/files/mcu761/files/guidelines_for_clinical_research.pdf>. Accessed 18 June 2020.

Harvard Medical School. (1999). Authorship Guidelines. <https://ari.hms.harvard.edu/sites/g/files/mcu761/files/authorship_guidelines.pdf>. Accessed 18 June 2020.

Harvey, S. (1994). Application of the CPA code of ethics in planning field research: An organizational case. *Canadian Psychology/Psychologie Canadienne*, 35(2), 204–219.

Hendrickson, T. L. (2015). Integrating responsible conduct of research education into undergraduate biochemistry and molecular biology laboratory curricula. *Biochemistry and molecular biology education: a bimonthly publication of the International Union of Biochemistry and Molecular Biology*, 43(2), 68–75. <https://doi.org/10.1002/bmb.20857>.

Hiney, M. (2015). Briefing Paper on Research Integrity: What it Means, Why it Is Important and How we Might Protect it. Science Europe: Science Europe Working Group on Research Integrity. <https://www.scienceeurope.org/our-resources/briefing-paper-on-research-integrity-what-it-means-why-it-is-important-and-how-we-might-protect-it>. Accessed 16 June 2020.

Holaday, M., & Yost, T. E. (1995). Authorship Credit and Ethical Guidelines. *Counseling and Values*, 40(1), 24–31.

Human Sciences Research Council (HSRC). (2006). Code of Research Ethics. <http://www.hsrc.ac.za/en/about/research-ethics>. Accessed 16 June 2020.

Idänpään-Heikkilä, J. E. (1994). WHO guidelines for good clinical practice (GCP) for trials on pharmaceutical products: responsibilities of the investigator. *Annals of Medicine*, 26(2), 89–94. <https://doi.org/10.3109/07853899409147334>.

International Association for Dental Research (IADR). (2009). Code of Ethics. <https://www.iadr.org/IADR/About-Us/Who-We-Are/Code-of-Ethics>. Accessed 16 June 2020.

International Council for Science (ICSU) (2014). Freedom, Responsibility and Universality of Science. <https://council.science/publications/freedom-responsibility-and-universality-of-science-2014/>. Accessed 16 June 2020.

Irish Universities Association (IUA). (2014). National Policy Statement on Ensuring Research Integrity in Ireland. <https://www.iua.ie/publications/national-policy-statement-on-ensuring-research-integrity-in-ireland/>. Accessed 19 June 2020.

Israel, M., & Drenth, P. (2016). Research Integrity: Perspectives from Australia and Netherlands. In T. Bretag (Ed.), Handbook of Academic Integrity (pp. 789–808). Singapore: Springer.

Komesaroff, P. A.(2005). Ethical issues in the relationships with industry: an ongoing challenge. New Guidelines open for public comment. *Journal of Paediatrics and Child Health*, 41(11), 558–560. <https://doi.org/10.1111/j.1440-1754.2005.00719.x>.

Korenman, S. G. (2006). Teaching the Responsible Conduct of Research in Humans (RCRH). <https://ori.hhs.gov/education/products/ucla/default.htm>. Accessed 18 June 2020.

Kreissl Lonfat, B. M., Kaufmann, I. M., & Rühli, F. (2015).A Code of Ethics for Evidence‐Based Research With Ancient Human Remains. *The Anatomical Record*, 298(6), 1175–1181. <https://doi.org/10.1002/ar.23126>.

Kyoto University. (2015). Promoting Research Integrity Regulations of Kyoto University. <https://www.kyoto-u.ac.jp/en/research/research-compliance-ethics/research-integrity-rules-reporting/documents/research-integrity-regulations201503.pdf>. Accessed 16 June 2020.

Kyoto University. (2018). Handbook on Research Fund Use. <http://www.kyoto-u.ac.jp/ja/research/rule/public/competitive/documents/handbook2018_eng.pdf>. Accessed 18 June 2020.

Levy, C., Rybak, A., Cohen, R., & Jung, C. (2017). [The Jardé law, a real simplification of research in France?]. *Archives de Pédiatrie*, 24(6), 571–577.

Lo, B., & Field, M. J. (Eds.). (2009). *Conflict of Interest in Medical Research, Education, and Practice*. Washington, DC: The National Academies Press.

Macrina, F. L. (2007). Scientific societies and promotion of the responsible conduct of research: codes, policies, and education. *Academic medicine: journal of the Association of American Medical Colleges*, 82(9), 865–869. <https://doi.org/10.1097/ACM.0b013e31812f7e58>.

Marušić, A. (2019). European Commission. Research and Innovation Observatory-Horizon 2020 Policy Support Facility. MLE on Research Integrity: Thematic Report No 3 - Dialogue and Communication. <https://rio.jrc.ec.europa.eu/policy-support-facility/mle-research-integrity>. Accessed 16 June 2020.

Maschke, K.J. (2003). US and UK policies governing research with humans. *Psychopharmacology*, 171, 47–55. <https://doi.org/10.1007/s00213-003-1666-9>.

Matheson, A. (2016). The ICMJE Recommendations and pharmaceutical marketing – strengths, weaknesses and the unsolved problem of attribution in publication ethics. *BMC Medical Ethics* 17, 20 (2016). <https://doi.org/10.1186/s12910-016-0103-7>.

Matheson, A. (2019). Can self-regulation deliver an ethical commercial literature? A critical reading of the “Good Publication Practice” (GPP3) guidelines for industry-financed medical journal articles. *Accountability in Research*, 26(2), 85–107. <https://doi.org/10.1080/08989621.2018.1564663>.

McIntosh, N., Bates, P., Brykczynska, G., Dunstan, G., Goldman, A., Harvey, D. *et al*. (2000). Guidelines for the ethical conduct of medical research involving children. Royal College of Paediatrics, Child Health: Ethics Advisory Committee. *Archives of Disease in Childhood*, 82(2), 177–182. <https://doi.org/10.1136/adc.82.2.177>.

Medical University of Graz. (2014). Standards of Good Scientific Practice and Ombuds Committee at the Medical University of Graz.

<https://www.medunigraz.at/en/qualitaetsmanagement-in-der-forschung/good-scientific-practice/>. Accessed 16 June 2020.

Medical University Vienna. (2013). Good Scientific Practice: Ethics in Science and Research. <https://www.meduniwien.ac.at/web/en/research/service-for-researchers/law-ethics/>. Accessed 18 June 2020.

Montreal Statement on Research Integrity in Cross-Boundary Research Collaborations. (2013). <https://wcrif.org/montreal-statement/file>. Accessed 19 June 2020.

Morris, S. E. (2010). Cracking the Code: Assessing Institutional Compliance with the Australian Code for the Responsible Conduct of Research. *Australian Universities’ Review*, 52(2), 18–26. <https://www.aur.org.au/archive/2010s>. Accessed 18 June 2020.

Nanyang Technological University (NTU). (2008). NTU Research Integrity Policy and Procedures. <https://www3.ntu.edu.sg/Research2/ResearchIntegrityPolicy.pdf>. Accessed 18 June 2020.

National Academies of Sciences, Engineering, and Medicine (NASEM). (2017). Fostering Integrity in Research. Washington, DC: The National Academies Press. <https://doi.org/10.17226/21896>.

National Academies of Sciences, Engineering, and Medicine (NASEM). (2018a). Open Science by Design: Realizing a Vision for 21st Century Research. Washington, DC: The National Academies Press. <https://doi.org/10.17226/25116>.

National Academies of Sciences, Engineering, and Medicine (NASEM). (2018b). *The Next Generation of Biomedical and Behavioral Sciences Researchers: Breaking Through*. Washington, DC: The National Academies Press. <https://doi.org/10.17226/25008>.

National Academies of Sciences, Engineering, and Medicine (NASEM). (2019). *Reproducibility and Replicability in Science*. Washington, DC: The National Academies Press. <https://doi.org/10.17226/25303>.

National Academy of Sciences, National Academy of Engineering, and Institute of Medicine. (1992). *Responsible Science: Ensuring the Integrity of the Research Process: Volume I.* Washington, DC: The National Academies Press. <https://doi.org/10.17226/1864>.

National Academy of Sciences, National Academy of Engineering, and Institute of Medicine. (1993). *Responsible Science: Ensuring the Integrity of the Research Process: Volume II*. Washington, DC: The National Academies Press. <https://doi.org/10.17226/2091>.

National Academy of Sciences, National Academy of Engineering, and Institute of Medicine. (2009a). Ensuring the Integrity, Accessibility, and Stewardship of Research Data in the Digital Age. Washington, DC: The National Academies Press. <https://doi.org/10.17226/12615>.

National Academy of Sciences, National Academy of Engineering, and Institute of Medicine. (2009b). *On Being a Scientist: A Guide to Responsible Conduct in Research: Third Edition*. Washington, DC: The National Academies Press. <https://doi.org/10.17226/12192>.

National Health and Medical Research Council (NHMRC), Australian Research Council, & Universities Australia. (2019a). *Authorship: A guide supporting the Australian Code for the Responsible Conduct of Research*. Canberra: National Health and Medical Research Council. <https://www.nhmrc.gov.au/about-us/publications/australian-code-responsible-conduct-research-2018>. Accessed 19 June 2020.

National Health and Medical Research Council (NHMRC), Australian Research Council, & Universities Australia. (2019b). *Disclosure of interest and management of conflicts of interest: A guide supporting the Australian Code for the Responsible Conduct of Research*. Canberra: National Health and Medical Research Council.

<https://www.nhmrc.gov.au/about-us/publications/australian-code-responsible-conduct-research-2018>. Accessed 19 June 2020.

National Health and Medical Research Council (NHMRC), Australian Research Council, & Universities Australia. (2019c). *Management of Data and Information in Research: A guide supporting Australian Code for the Responsible Conduct of Research*. Canberra: National Health and Medical Research Council. <https://www.nhmrc.gov.au/about-us/publications/australian-code-responsible-conduct-research-2018>. Accessed 19 June 2020.

National Health and Medical Research Council (NHMRC), Australian Research Council, & Universities Australia. (2019d). *Peer Review: A guide supporting the Australian Code for Responsible Conduct of Research*. Canberra: National Health and Medical Research Council. <https://www.nhmrc.gov.au/about-us/publications/australian-code-responsible-conduct-research-2018>. Accessed 19 June 2020.

National Health Research Ethics Committee (NHREC). (2007). *National Code of Health Research Ethics*. Abuja: Federal Ministry of Health. <http://www.nhrec.net/nhrec/NCHRE_Aug%2007.pdf>. Accessed 17 June 2020.

National Institutes of Health (NIH). (2003). NIH Data Sharing Policy and Implementation Guidance. <https://grants.nih.gov/grants/policy/data_sharing/data_sharing_guidance.htm>. Accessed 18 June 2020.

National Institutes of Health (NIH). (2011). Update on the Requirement for Instruction in the Responsible Conduct of Research. <https://grants.nih.gov/grants/guide/notice-files/not-od-10-019.html>. Accessed 17 June 2020.

Nature Research [Internet]. Editorial policies. <https://www.nature.com/nature-research/editorial-policies>. Accessed 19 June 2020.

Nebeker, C., & López-Arenas, A. (2016). Building Research Integrity and Capacity (BRIC): An Educational Initiative to Increase Research Literacy among Community Health Workers and Promotores. *Journal of Microbiology & Biology Education*, 17(1), 41–45. <https://doi.org/10.1128/jmbe.v17i1.1020>.

Nepal Health Research Council (NHRC). (2011). *National Ethical Guidelines For Health Research in Nepal And Standard Operating Procedures*. Ramshah Path: Nepal Health Research Council. <http://nhrc.gov.np/wp-content/uploads/2017/02/National_Ethical_Guidelines.pdf>. Accessed 17 June 2020.

Netherlands Code of Conduct for Research Integrity. (2018).

<https://www.nwo.nl/en/policies/scientific+integrity+policy/netherlands+code+of+conduct+for+research+integrity>. Accessed 17 June 2020.

Nichols-Casebolt, A. (2012). *Research Integrity and Responsible Conduct of Research*. New York, NY: Oxford University Press, Inc.

Northern Illinois University. (2006a). Peer Review Quick Guide: Detecting Common Mistakes and Considering Dilemmas in Peer Review. [https://ori.hhs.gov/education/products/niu_peerreview/#](https://ori.hhs.gov/education/products/niu_peerreview/). Accessed 19 June 2020.

Northern Illinois University. (2006b). Responsible Authorship Quick Guide: Detecting Common Mistakes and Considering Dilemmas in Responsible Authorship. [https://ori.hhs.gov/education/products/niu_authorship/index.htm#](https://ori.hhs.gov/education/products/niu_authorship/index.htm). Accessed 19 June 2020.

Norwegian Institute of Biomedical Science (NITO). (2006). Ethics for Biomedical Laboratory Scientists: Professional Ethical Guidelines for Biomedical Laboratory Scientists. <https://www.nito.no/contentassets/7152ab4936194074b7b10d18500bcfa7/ethics-for-biomedical-laboratory-scientists.pdf>. Accessed 19 June 2020.

Nuffield Council on Bioethics. (1995). Human Tissue Ethical and Legal Issues. London: Nuffield Council on Bioethics. <https://www.nuffieldbioethics.org/publications/human-tissue>. Accessed 19 June 2020.

Nys, H. (2012). New European Rules Regarding the Approval of Clinical Trials, the Role of Ethics Committees and the Protection of Subjects. *Archivum immunologiae et therapiae experimentalis*, 60(6), 405–414. <https://doi.org/10.1007/s00005-012-0200-3>.

Panacek, E. A., & Lewis, R. J. (1995). Guidelines for clinical investigator involvement in industry-sponsored clinical trials. SAEM Research Committee. Academic emergency medicine : *Official Journal of the Society for Academic Emergency Medicine*, 2(1), 43–45. <https://doi.org/10.1111/j.1553-2712.1995.tb03081.x>.

Parder, M., & Juurik M. (2019). Promoting ethics and integrity in non-medical research (PRO-RES) project. Reporting on existing Codes and Guidelines. <http://prores-project.eu/wp-content/uploads/2019/12/D1_Existing_Code_and_guidelines.pdf>. Accessed 17 June 2020.

Path2Integrity project [Internet]. Teaching Research Integrity and Research Ethics. <https://www.path2integrity.eu/teaching-RI>. Accessed 19 June 2020.

Penders, B., Shaw, D., Lutz, P., Townend, D., Akrong, L., & Zvonareva, O. (2018). ENERI Manual: Research Integrity and Ethics. <http://eneri.eu/reri-manual/>. Accessed 18 June 2020.

Pharmaceutical Research and Manufacturers of America (PhRMA). (2014). Principles on Conduct of Clinical Trials: Communication of Clinical Trials Results. Washington, DC: Pharmaceutical Research and Manufacturers of America. <https://www.phrma.org/en/Codes-and-guidelines/PhRMA-Principles-on-Conduct-of-Clinical-Trials>. Accessed 17 June 2020.

Phillips, M. S. (1999). Clinical research: ASHP Guidelines and Future Directions for Pharmacists. *American Journal of Health-System Pharmacy: AJHP: Official Journal of the American Society of Health-System Pharmacists*, 56(4), 344–346. <https://doi.org/10.1093/ajhp/56.4.344>.

Queen's University Belfast (QUB). (2014). QUB Code of Conduct and Integrity in Research. <https://www.qub.ac.uk/Research/Governance-ethics-and-integrity/Policies-procedures-and-guidelines/>. Accessed 19 June 2020.

Resnik, D. B., & Shamoo, A. E. (2011). The Singapore Statement on Research Integrity. Accountability in research, 18(2), 71–75. <https://doi.org/10.1080/08989621.2011.557296>.

Rockwell, S. (2014). Ethics of Peer Review: A Guide for Manuscript Reviewers. <https://ori.hhs.gov/sites/default/files/prethics.pdf>. Accessed 19 June 2020.

Roig, M. (2015). Avoiding plagiarism, self-plagiarism, and other questionable writing practices: A guide to ethical writing. <https://ori.hhs.gov/sites/default/files/plagiarism.pdf>. Accessed 19 June 2020.

Royal College of Nursing (RCN). (2004). *Research ethics: RCN guidance for nurses*. London: Royal College of Nursing. <https://rcn.access.preservica.com/uncategorized/IO_86f61e16-bbb6-405d-a499-0ae5ac56ce62/>. Accessed 19 June 2020.

Royal College of Physicians. (2007). *Guidelines on the practice of ethics committees in medical research with human participants (4^th^ ed.)*. London: Royal College of Physicians.

Royal Netherlands Academy of Arts and Sciences (KNAW). (2008). *A Code of Conduct for Biosecurity: Report by the Biosecurity Working Group*. Amsterdam: Royal Netherlands Academy of Arts.

Royal Netherlands Academy of Arts and Sciences (KNAW). (2013). *Responsible Research Data Management and the Prevention of Scientific Misconduct*. Amsterdam: Royal Netherlands Academy of Arts and Sciences.

San Francisco Declaration on Research Assessment (DORA). (2012). <https://sfdora.org/read/>. Accessed 19 June 2020.

Santos, J., Palumbo, F., Molsen-David, E., Willke, R. J., Binder, L., Drummond, M. *et al.* (2017). ISPOR Code of Ethics 2017 (4th Edition). *Value in health: the journal of the International Society for Pharmacoeconomics and Outcomes Research*, 20(10), 1227–1242. <https://doi.org/10.1016/j.jval.2017.10.018>.

Saver, R. S. (2014). Shadows amid sunshine: regulating financial conflicts in medical research. *Chest*, 145(2), 379–385. <https://doi.org/10.1378/chest.13-1719>.

Science Council of Japan (SCJ). (2006). Code of Conduct for Scientists. <http://www.scj.go.jp/en/report/code.html>. Accessed 19 June 2020.

Science Europe. (2018a). Guidance document: Presenting a Framework for Discipline-specific

Research Data Management. <http://www.scienceeurope.org/our-resources/guidance-document-presenting-a-framework-for-discipline-specific-research-data-management>. Accessed 17 June 2020.

Science Europe. (2018b). Practical Guide to the International Alignment of Research Data Management. <https://www.scienceeurope.org/our-resources/practical-guide-to-the-international-alignment-of-research-data-management/>. Accessed 17 June 2020.

South African Medical Research Council (SAMRC). (2018). The South African Medical Research Council Guidelines on the Responsible Conduct of Research. <https://www.samrc.ac.za/research/ethics/guideline-documents>. Accessed 17 June 2020.

Spanish National Research Council (CSIC). (2015). CSIC Manual of Conflict of interest. <http://www.cnb.csic.es/documents/ConflictosInteresCSIC.pdf>. Accessed 19 June 2020.

Spanish National Research Council (CSIC). (2015). National Statement on Scientific Integrity. <http://www.enrio.eu/wp-content/uploads/2017/03/csic-national-statement-on-scientific-integrity.pdf>. Accessed 17 June 2020.

Swiss Academies of Arts and Sciences. (2013). Authorship in scientific publications - Analysis and recommendations. <http://www.akademien-schweiz.ch/en/index/Publikationen/Archiv/Richtlinien-Empfehlungen.html>. Accessed 19 June 2020.

The National Committee for Research Ethics in Science and Technology (NENT). (2016). *Guidelines for Research Ethics in Science and Technology (2nd ed.)*. Oslo: The Norwegian National Research Ethics Committees. <https://www.etikkom.no/en/ethical-guidelines-for-research/guidelines-for-research-ethics-in-science-and-technology/>. Accessed 19 June 2020.

The National Committee for Research Ethics in Science and Technology (NENT). (2018). *Ethical Guidelines for the Use of Animals in Research*. Oslo: The Norwegian National Research Ethics Committees. <https://www.etikkom.no/globalassets/documents/publikasjoner-som-pdf/etiske-retningslinjer-for-bruk-av-dyr-i-forskning/ethical-guidelines-for-the-use-of-animals-in-research.pdf>. Accessed 19 June 2020.

The National Committee for Research Ethics in Social Sciences and the Humanities (NESH). (2016). *Guidelines for Research Ethics in the Social Sciences, Humanities, Law and Theology (4th ed.)*. Oslo: The Norwegian National Research Ethics Committees. <https://www.etikkom.no/en/ethical-guidelines-for-research/guidelines-for-research-ethics-in-the-social-sciences--humanities-law-and-theology/>. Accessed 19 June 2020.

The National Committee for Research on Human Remains. (2016). *Guidelines for Research Ethics on Human Remains*. Oslo: The Norwegian National Research Ethics Committees. <https://www.etikkom.no/en/ethical-guidelines-for-research/guidelines-for-research--ethics-on-human-remains/>. Accessed 19 June 2020.

Toom, K., & Miller, P. F. (2018). Ethics and Integrity. In: J. Andersen, K. Toom, & S. Poli, *Research Management: Europe and Beyond* (pp. 264–286). London: Academic Press, Elsevier Inc.

TRUST project. (2018). Global Code of Conduct for Research in Resource-Poor Settings. 2018. <https://www.globalcodeofconduct.org/>. Accessed 17 June 2020.

United Kingdom Health Ministers’ Gene Therapy Advisory Committee. (1995). Guidance on making proposals to conduct gene therapy research on human subjects. Report of the United Kingdom Health Ministers' Gene Therapy Advisory Committee. (1995). *Human Gene Therapy*, 6(3), 335–346. <https://doi.org/10.1089/hum.1995.6.3-335>.

United Kingdom Research Integrity Office (UKRIO). (2009). Code of Practice for Research: Promoting good practice and preventing misconduct. <https://ukrio.org/wp-content/uploads/UKRIO-Code-of-Practice-for-Research.pdf>. Accessed 17 June 2020.

United Nations Educational, Scientific and Cultural Organization (UNESCO). (2004). Code of conduct social science research. <http://www.unesco.org/new/fileadmin/MULTIMEDIA/HQ/SHS/pdf/Soc_Sci_Code.pdf>. Accessed 19 June 2020.

Universities UK. (2019). The Concordat to Support Research Integrity. <https://www.universitiesuk.ac.uk/policy-and-analysis/reports/Documents/2019/the-concordat-to-support-research-integrity.pdf>. Accessed 18 June 2020.

University of Alabama at Birmingham. (2008). Online Learning Tool for Research Integrity and Image Processing. <https://ori.hhs.gov/education/products/RIandImages/default.html>. Accessed 19 June 2020.

University of Connecticut. (2011). Code of Conduct. <https://policy.uconn.edu/2011/05/17/employee-code-of-conduct/#research>. Accessed 19 June 2020.

University of Oxford. Policy on the Management of Data Supporting Research Outputs [Internet]. <https://researchdata.ox.ac.uk/university-of-oxford-policy-on-the-management-of-data-supporting-research-outputs/>. Accessed 18 June 2020.

University of Oxford. (2018a). Code of practice and procedure on academic integrity in research. <https://hr.admin.ox.ac.uk/academic-integrity-in-research#collapse1316006>. Accessed 18 June 2020.

University of Oxford. (2018b). Data protection policy.

<https://compliance.admin.ox.ac.uk/data-protection-policy#collapse1172256>. Accessed 18 June 2020.

University of Oxford. (2019a). Conflict of interest policy. <https://researchsupport.admin.ox.ac.uk/governance/integrity/conflict/policy>. Accessed 18 June 2020.

University of Oxford. (2019b). Policy on the ethical research involving human participants and personal data. <https://researchsupport.admin.ox.ac.uk/governance/ethics/committees/policy#collapse395121>. Accessed 18 June 2020.

University of Southern Queensland (USQ). (2018). Animal Ethics Committee Procedure. <https://policy.usq.edu.au/documents/141878PL>. Accessed 18 June 2020.

University of Southern Queensland (USQ). (2019). Conflict of Interest Policy. <https://policy.usq.edu.au/documents/142758PL>. Accessed 18 June 2020.

University of Southern Queensland (USQ). (2020). Authorship Procedure. <https://policy.usq.edu.au/documents/142211PL>. Accessed 19 June 2020.

University of Tartu, Centre for Ethics, & Estonian Research Council. (2017). Estonian Code of Conduct for Research Integrity. Tartu: Centre for Ethics, University of Tartu. <https://www.eetika.ee/sites/default/files/www_ut/hea_teadustava_eng_trukis.pdf>. Accessed 18 June 2020.

University of Utrecht. (2014). Academic Integrity Checklist. <https://students.uu.nl/sites/default/files/uu-academicintegrity.pdf>. Accessed 18 June 2020.

University of Waikato. (2008). Ethical Conduct in Human Research and Related Activities Regulation. <https://calendar.waikato.ac.nz/assessment/ethicalConduct.html>. Accessed 19 June 2020.

University of Wollongong. (2017). Authorship Policy. <https://documents.uow.edu.au/about/policy/uow058654.html>. Accessed 19 June 2020.

Wager, E., & Kleinert, S. (2011). Responsible research publication: international standards for authors. A position statement developed at the 2^nd^ World Conference on Research Integrity (Singapore, July 22-24, 2010). In: T. Mayer, & N. Steneck (Eds.), *Promoting Research Integrity in a Global Environment* (pp. 309–316). Singapore: Imperial College Press, World Scientific Publishing.

Wellcome Trust. (2018). Good research practice guidelines. <https://wellcome.ac.uk/grant-funding/guidance/good-research-practice-guidelines>. Accessed 18 June 2020.

World Economic Forum (WLF). (2018). Code of Ethics. <http://www3.weforum.org/docs/WEF_Code_of_Ethics.pdf>. Accessed 18 June 2020.

World Medical Association (WMA). (2018). WMA Declaration of Helsinki - Ethical Principles for Medical Research Involving Human Subjects. <https://www.wma.net/policies-post/wma-declaration-of-helsinki-ethical-principles-for-medical-research-involving-human-subjects/>. Accessed 18 June 2020.
