# Supplementary material for: Practices for Research Integrity Promotion in Research Performing Organisations and Research Funding Organisations: A Scoping Review
Source: Sci Eng Ethics. 2021 Jan 27;27(1):4. doi: 10.1007/s11948-021-00281-1 (PMC7840650; doi:10.1007/s11948-021-00281-1)
Supplement: Supplementary file 6 — Supplementary material 6 (DOCX 23 kb) [file 11948_2021_281_MOESM6_ESM.docx]

**Appendix 6 Comparison of fundamental principles from the ALLEA code and the NASEM – *Fostering Integrity in Research* and matching principles found in other documents**

| **Principles from European Code of Conduct for RI** | **Principles from the NASEM *Fostering Integrity in Research* book** | **Matching principles identified in other documents** |
| --- | --- | --- |
| **Reliability** (Ensuring the quality of research by proper use of methodology, analysis and resources) (ALLEA 2017) | **Accountability** (Being able to demonstrate the validity of research which will be possible by using a proper methodology) (NASEM 2017) | **Balance** (Wager and Kleinert 2011)  **Critical, open-minded approach** (ESF 2000)  **Essentiality** (CEHAT 2000)  **Excellence** (UKRIO 2009)  **High professional standards** (ESF 2000)  **Knowledge, ability and commitment to do research** (CEHAT 2000)  **Professional competence** (ASA 2018)  **Professional integrity** (IADR 2009)  **Quality and rigour** (EECERA 2015)  **Reliability** (IAC and IAP 2012; Association of Universities in the Netherlands 2012; Wager and Kleinert 2011; IUA 2014; University of Utrecht 2014)  **Research merit** (NHMRC 2018b)  **Rigour** (NHMRC 2018a; Universities UK 2019)  **Scientific and academic professionalism** (HSRC 2006)  **Scrupulousness** (University of Utrecht 2014; Netherlands Code of Conduct for Research Integrity 2018)  **Scepticism** (IAC and IAP 2012)  **Soundness** (Wager and Kleinert 2011)  **Training and skills** (UKRIO 2009)  **Verifiability** (Association of Universities in the Netherlands 2012) |
| **Honesty** (Being honest, fair and transparent in developing, conducting, evaluating and reporting research) (ALLEA 2017) | **Honesty** (Honesty is a prerequisite of good research and other principles) (NASEM 2017)  **Objectivity** (Researchers' independence in performing research, avoidance of pressure and biases to be able to present research results truthfully) (NASEM 2017)  **Openness** (Being transparent in all researchers phases, presenting all relevant information to other researchers, research participants and society) (NASEM 2017) | **Balance** (Wager and Kleinert 2011)  **Communication** (Montreal Statement 2013; IUA 2014)  **Cooperation** (UKRIO 2009; University of Tartu 2017)  **Fairness** (IUA 2014; TRUST 2018)  **Freedom** (University of Tartu 2017)  **Honesty** (ESF 2000; IADR 2009; UKRIO 2009; Resnik and Shamoo 2011; IAC and IAP 2012; Wager and Kleinert 2011; Danish Ministry of Higher Education and Science 2014; IUA 2014; University of Tartu 2017; Netherlands Code of Conduct for Research Integrity 2018; NHMRC 2018a; TRUST 2018; Universities UK 2019)  **Impartiality** (Association of Universities in the Netherlands 2012; University of Utrecht 2014; IUA 2014)  **Independence** (Association of Universities in the Netherlands 2012; IUA 2014; Netherlands Code of Conduct for Research Integrity 2018)  **Integrity** (UKRIO 2008; UKRIO 2009; Montreal Statement 2013; EECERA 2015; ASA 2018; NHMRC 2018a)  **Justice** (NHMRC 2018a)  **Objectivity** (IADR 2009; IAC and IAP 2012; IUA 2014; University of Tartu, 2017)  **Openness** (IAC and IAP 2012; University of Tartu 2017; Moher et al. 2019)  **Originality** (Wager and Kleinert 2011)  **Respectful interactions** (EECERA 2015)  **Scrupulousness** (Association of Universities in the Netherlands 2012)  **Transparency** (CEHAT 2000; HSRC 2006; Wager and Kleinert 2011; Montreal Statement 2013; Danish Ministry of Higher Education and Science 2014; EECERA 2015; NHMRC 2018a; Moher et al. 2019; Universities UK 2019) |
| **Respect** (Respecting colleagues, research participants, society and environment) (ALLEA 2017) | **Stewardship** (Good stewardship toward other researchers, organisation and science overall) (NASEM 2017)  **Fairness** (Being fair in research evaluation or toward research participants and animals when conducting research; acknowledging the work of others fairly) (NASEM 2017) | **Appropriate authorship and acknowledgements** (Wager and Kleinert 2011)  **Balance** (UKRIO 2008)  **Beneficence** (CEHAT 2000; CIOMS and WHO 2009; NHRC 2011; Santos et al. 2017; NHMRC 2018b)  **Care** (IUA 2014; University of Tartu 2017; TRUST 2018; Universities UK 2019)  **Collaboration** (University of Utrecht 2014)  **Consideration** (IADR 2009)  **Equity** (EECERA 2015)  **Fairness** (ESF 2000; UKRIO 2008; IAC and IAP 2012; NHMRC 2018a)  **Frankness** (ESF 2000)  **Goals** (Montreal Statement 2013)  **Honour** (IADR, 2009)  **Inclusiveness** (Moher et al. 2019)  **Integrity** (ASA 2018)  **Justice** (NHRC 2011; EECERA 2015; Santos et al. 2017; University of Tartu 2017)  **Knowing multiple perspectives** (EECERA 2015)  **Maximisation of public interest and social justice** (CEHAT 2000)  **Non-exploitation** (CEHAT 2000)  **Non-maleficence** (CEHAT 2000)  **Precaution and risk minimisation** (CEHAT 2000)  **Professional courtesy** (Resnik and Shamoo 2011)  **Promotion** (NHMRC 2018a)  **Prudence** (IADR 2009)  **Recognition** (NHMRC 2018a)  **Respect (people's rights, dignity, diversity, democratic values, the autonomy of research participants, environment, privacy, anonymity and confidentiality)** (CEHAT 2000; HSRC 2006; UKRIO 2008; CIOMS and WHO 2009; NHRC 2011; EECERA 2015; Santos et al. 2017; University of Tartu 2017; ASA 2018; NHMRC 2018a; NHMRC 2018b; TRUST 2018; Universities UK 2019)  **Responsible reporting** (Wager and Kleinert 2011)  **Safety** (UKRIO 2009) |
| **Accountability** (Researchers and research organisations are responsible for their research and its impact, mentoring, education and training) (ALLEA 2017) | **Accountability** (Being accountable for research behaviour, work and actions; researchers have an obligation to explain the validity of their work, as well as the responsibility of being trustworthy toward organisation and society. Funders are accountable for evaluating research proposals and providing grants) (NASEM 2017) | **Accountability** (CEHAT 2000; HSRC 2006; UKRIO 2009; Resnik and Shamoo 2011; IAC and IAP 2012; Wager and Kleinert 2011; Danish Ministry of Higher Education and Science 2014; NHMRC 2018a; Universities UK 2019)  **Contributing to societal needs** (Moher et al. 2019)  **Prevention of detriment** (UKRIO 2008)  **Public domain** (CEHAT 2000)  **Purpose** (Montreal Statement 2013)  **Responsibility** (Wager and Kleinert 2011; IUA 2014; University of Tartu 2017; Netherlands Code of Conduct for Research Integrity 2018; Moher et al. 2019)  **Resource management** (Montreal Statement 2013)  **Roles and responsibilities** (Montreal Statement 2013)  **Social contribution** (EECERA 2015)  **Social responsibility** (ASA 2018)  **Totality of responsibility** (CEHAT 2000) |

ASA – American Sociological Association; CEHAT – Centre for Enquiry into Health and Allied Themes; CIOMS – Council for International Organizations of Medical Sciences; EECERA – European Early Childhood Education Research Association; ESF – European Science Foundation; HSRC – Human Sciences Research Council; IAC – Inter Academy Council; IADR – International Association for Dental Research; IAP – Inter Academy Partners; NHMRC – National Health and Medical Research Council (Australia); NHRC – Nepal Health Research Council; RI – research integrity; UK – United Kingdom; UKRIO – United Kingdom Research Integrity Office; WHO – World Health Organization

**References**

All European Academies (ALLEA). (2017). European Code of Conduct for Research Integrity. <https://allea.org/code-of-conduct/>. Accessed 17 June 2020.

American Sociological Association (ASA). (2018). Code of Ethics. <https://www.asanet.org/code-ethics>. Accessed 17 June 2020.

Association of Universities in the Netherlands (VSNU). (2012). The Netherlands Code of Conduct for Scientific Practice: Principles of good scientific teaching and research. <https://www.vsnu.nl/files/documenten/Domeinen/Onderzoek/The_Netherlands_Code_of_Conduct_for_Scientific_Practice_2012.pdf>. Accessed 15 June 2020.

Centre for Enquiry into Health and Allied Themes (CEHAT). (2000). National Committee for Ethics in Social Science Research in Health (NCESSRH): Ethical Guidelines for Social Science Research in Health. <http://www.cehat.org/go/uploads/EthicalGuidelines/ethicalguidelines.pdf>. Accessed 15 June 2020.

Council for International Organizations of Medical Sciences (CIOMS), & World Health Organization (WHO). (2009). International Ethical Guidelines for Epidemiological Studies.

<https://cioms.ch/wp-content/uploads/2017/01/International_Ethical_Guidelines_LR.pdf>. Accessed 15 June 2020.

Danish Ministry of Higher Education and Science. (2014). Danish Code of Conduct for Research Integrity. <https://ufm.dk/en/publications/2014/files-2014-1/the-danish-code-of-conduct-for-research-integrity.pdf>. Accessed 17 June 2020.

European Early Childhood Education Research Association (EECERA). (2015). EECERA Ethical Code for Early Childhood Researchers. <https://www.eecera.org/wp-content/uploads/2016/07/EECERA-Ethical-Code.pdf>. Accessed 15 June 2020.

European Science Foundation (ESF). (2000). Good scientific practice in research and scholarship. <http://archives.esf.org/fileadmin/Public_documents/Publications/ESPB10.pdf>. Accessed 16 June 2020.

Human Sciences Research Council (HSRC). (2006). Code of Research Ethics. <http://www.hsrc.ac.za/en/about/research-ethics>. Accessed 16 June 2020.

Inter Academy Council (IAC), & Inter Academy Partners (IAP). (2012). Responsible Conduct in the Global Research Enterprise: A Policy Report. <https://www.interacademies.org/publication/responsible-conduct-global-research-enterprise>. Accessed 17 June 2020.

International Association for Dental Research (IADR). (2009). Code of Ethics. <https://www.iadr.org/IADR/About-Us/Who-We-Are/Code-of-Ethics>. Accessed 16 June 2020.

Irish Universities Association (IUA). (2014). National Policy Statement on Ensuring Research Integrity in Ireland. <https://www.iua.ie/publications/national-policy-statement-on-ensuring-research-integrity-in-ireland/>. Accessed 17 June 2020.

Moher, D., Bouter, L., Kleinert, S., Glasziou, P., Sham, M. H., Barbour, V. et al. (2019). The Hong Kong Principles for Assessing Researchers: Fostering Research Integrity. <https://doi.org/10.31219/osf.io/m9abx>. Accessed 17 June 2020.

Montreal Statement on Research Integrity in Cross-Boundary Research Collaborations. (2013). <https://wcrif.org/montreal-statement/file>. Accessed 17 June 2020.

National Academies of Sciences, Engineering, and Medicine (NASEM). (2017). *Fostering Integrity in Research*. Washington, DC: The National Academies Press. <https://doi.org/10.17226/21896>. Accessed 17 June 2020.

National Health and Medical Research Council (NHMRC), Australian Research Council, & Universities Australia. (2018a). *Australian Code for the Responsible Conduct of Research*. Canberra: National Health and Medical Research Council.

<https://www.nhmrc.gov.au/about-us/publications/australian-code-responsible-conduct-research-2018>. Accessed 17 June 2020.

National Health and Medical Research Council (NHMRC), Australian Research Council, & Universities Australia. (2018b). *National Statement on Ethical Conduct in Human Research 2007 (updated 2018)*. Canberra: National health and Medical Research Council.

<https://www.nhmrc.gov.au/about-us/publications/national-statement-ethical-conduct-human-research-2007-updated-2018#block-views-block-file-attachments-content-block-1>. Accessed 17 June 2020.

Nepal Health Research Council (NHRC). (2011). *National Ethical Guidelines For Health Research in Nepal And Standard Operating Procedures*. Ramshah Path: Nepal Health Research Council. <http://nhrc.gov.np/wp-content/uploads/2017/02/National_Ethical_Guidelines.pdf>. Accessed 17 June 2020.

Netherlands Code of Conduct for Research Integrity. (2018). <https://www.nwo.nl/en/policies/scientific+integrity+policy/netherlands+code+of+conduct+for+research+integrity>. Accessed 17 June 2020.

Resnik, D. B., & Shamoo, A. E. (2011). The Singapore Statement on Research Integrity. *Accountability in research*, 18(2), 71–75. <https://doi.org/10.1080/08989621.2011.557296>.

Santos, J., Palumbo, F., Molsen-David, E., Willke, R. J., Binder, L., Drummond, M. *et al.* (2017). ISPOR Code of Ethics 2017 (4th Edition). *Value in health: the journal of the International Society for Pharmacoeconomics and Outcomes Research*, 20(10), 1227–1242. <https://doi.org/10.1016/j.jval.2017.10.018>.

TRUST project. (2018). Global Code of Conduct for Research in Resource-Poor Settings. 2018. <https://www.globalcodeofconduct.org/>. Accessed 17 June 2020.

United Kingdom Research Integrity Office (UKRIO). (2008). Procedure for the investigation of misconduct in research. <https://ukrio.org/wp-content/uploads/UKRIO-Procedure-for-the-Investigation-of-Misconduct-in-Research.pdf>. Accessed 17 June 2020.

United Kingdom Research Integrity Office (UKRIO). (2009). Code of Practice for Research: Promoting good practice and preventing misconduct. <https://ukrio.org/wp-content/uploads/UKRIO-Code-of-Practice-for-Research.pdf>. Accessed 17 June 2020.

Universities UK. (2019). The Concordat to Support Research Integrity. <https://www.universitiesuk.ac.uk/policy-and-analysis/reports/Documents/2019/the-concordat-to-support-research-integrity.pdf>. Accessed 18 June 2020.

University of Tartu, Centre for Ethics, & Estonian Research Council. (2017). Estonian Code of Conduct for Research Integrity. Tartu: Centre for Ethics, University of Tartu. <https://www.eetika.ee/sites/default/files/www_ut/hea_teadustava_eng_trukis.pdf>. Accessed 18 June 2020.

University of Utrecht. (2014). Academic Integrity Checklist. <https://students.uu.nl/sites/default/files/uu-academicintegrity.pdf>. Accessed 18 June 2020.

Wager, E., & Kleinert, S. (2011). Responsible research publication: international standards for authors. A position statement developed at the 2^nd^ World Conference on Research Integrity (Singapore, July 22-24, 2010). In: T. Mayer, & N. Steneck (Eds.), *Promoting Research Integrity in a Global Environment* (pp. 309–316). Singapore: Imperial College Press, World Scientific Publishing.
